# Supplementary material for: Non-invasive tests for fibrotic MASH for reducing screen failure in therapeutic trials
Source: JHEP Rep. 2025 Feb 4;7(4):101351. doi: 10.1016/j.jhepr.2025.101351 (PMC11985113; doi:10.1016/j.jhepr.2025.101351)

Reducing screen failure in MASH therapeutic trials using dedicated non-invasive tests for fibrotic MASH

Jeanne Fichez, Thomas Mouillot, Luisa Vonghia, Charlotte Costentin, Clémence Moreau, Marine Roux, Adèle Delamarre, Sven Francque, Ming-Hua Zheng, Jérôme Boursier

Table of contents

Table S1 2

Table S2 3

Table S3 4

Table S4 5

Table S5 6

Table S6 7

Table S7 8

Table S8 9

Table S9 10

Table S10 11

Fig. S1 12

Fig. S2 15

Fig. S3 17

Fig. S4 18

Table S1: Non-invasive tests evaluated in the study

Category		Test	Diagnostic target [a]	Variable
Blood tests	Simple	FNI (1)	Fibrotic MASH	AST, HbA1c, HDL cholesterol
		FIB4 (2)	Advanced fibrosis	Age, AST, ALT, platelets
	Specialized	MACK-3 (3)	Fibrotic MASH	AST, HOMA index, CK-18
		ELF (4)	Advanced fibrosis	Hyaluronate, TIMP1, P3NP
		Fibrotest (5)	Advanced fibrosis	Age, sex, gamma-GT, bilirubin, haptoglobin, apolipoproteinA1, α2macroglobulin
Elastography-based tests		VCTE (2)	-	Liver stiffness
		FAST (6)	Fibrotic MASH	AST, liver stiffness, CAP
		Agile3+ (7)	Advanced fibrosis	Age, sex, diabetes, AST, ALT, platelets, liver stiffness

[a] Diagnostic target for which the test was developed.

ALT: alanine aminotransferase; AST: aspartate aminotransferase; CAP: control attenuation parameter; CK-18: Cytokeratin 18; Gamma-GT: gamma-glutamyl transferase; HbA1c: hemoglobin A1C; HDL: high-density lipoprotein; HOMA index: homeostatic model assessment index; MASH: metabolic dysfunction - associated steatohepatitis; P3NP: procollagen type III N-terminal peptide; TIMP1: tissue inhibitor of metalloproteinase-1; VCTE: vibration controlled transient elastography (Fibroscan device, Echosens, Paris, France)

References

1. Tavaglione F, et al. Clin Gastroenterol Hepatol 2023;21:1523-1532 e1521.
2. Mozes FE, et al. Gut 2022;71:1006-1019.
3. Boursier J, et al. Aliment Pharmacol Ther 2018;47:1387-1396.
4. Vali Y, et al. J Hepatol 2020;73:252-262.
5. Vali Y, et al. J Clin Med 2021;10.
6. Newsome PN, et al. Lancet Gastroenterol Hepatol 2020;5:362-373.
7. Sanyal AJ, et al. J Hepatol 2023;78:247-259.

Table S2: Study analyses

Group	Patients (n)	Fibrosis tests available	Analyses
Study population	1,005	FNI, FIB4	• Direct comparison of two simple blood fibrosis tests: FNI (developed for fibrotic MASH) and FIB4 (developed for advanced fibrosis)
Elastography group	817	FNI, FIB4<br>VCTE<br>FAST, Agile3+	• Direct comparison of three elastography-based tests: VCTE, FAST (developed for fibrotic MASH), and Agile3+ (developed for advanced fibrosis)<br>• Evaluation of the added value of elastography-based tests (VCTE, FAST, Agile3+) compared to simple blood tests (FNI, FIB4)
Specialized blood tests group	545	FNI, FIB4<br>MACK-3<br>ELF, Fibrotest	• Direct comparison of three specialized blood fibrosis tests: MACK-3 (developed for fibrotic MASH), ELF (developed for advanced fibrosis), and Fibrotest (developed for advanced fibrosis)<br>• Evaluation of the added value of specialized blood tests (MACK-3, ELF, Fibrotest) compared to simple blood tests (FNI, FIB4)

Table S3: AUROC for the diagnosis of fibrotic MASH and the diagnosis of advanced fibrosis in the 747 patients with reliable VCTE

	Elastography group with reliable VCTE result (n = 747)	
**Diagnostic target:**	**Fibrotic MASH**	**Advanced fibrosis**
FNI	0.689 (0.651-0.727)	0.689 (0.648-0.730)
FIB4	0.663 (0.624-0.702)	0.792 (0.758-0.826)
FAST	0.759 (0.725-0.793)	0.759 (0.723-0.796)
VCTE	0.722 (0.685-0.759)	0.825 (0.794-0.857)
Agile3+	0.701 (0.664-0.739)	0.859 (0.831-0.859)
P value (comparison):		
FNI *vs.* FIB4	0.554	<0.001
FNI *vs.* FAST	<0.001	<0.001
FNI *vs.* VCTE	0.469	<0.001
FNI *vs.* Agile 3+	0.618	<0.001
FIB4 *vs.* FAST	<0.001	0.518
FIB4 *vs.* VCTE	0.056	0.647
FIB4 *vs.* Agile3+	0.061	<0.001
FAST *vs.* VCTE	0.108	<0.001
FAST *vs.* Agile3+	0.038	<0.001
Agile3+ *vs.* VCTE	0.551	0.047

MASH: metabolic dysfunction-associated steatohepatitis; VCTE: vibration controlled transient elastography

Table S4: Internal validation on 1,000 bootstrap samples of the optimized thresholds of FNI, FIB4, VCTE, Agile3+, ELF and Fibrotest for fibrotic MASH

Results correspond to mean percentages with 95% confidence intervals in parentheses.

Group	Test	Threshold	Patients (%) [a]	Se (%)	Spe (%)	NPV (%)	PPV (%)	Grey zone (%) [b]
Study population (n = 1,055)	FNI	<0.23	24.5 (24.4, 24.5)	89.8 (89.7, 89.8)	34.6 (34.6, 34.7)	82.5 (82.4, 82.6)	49.6 (49.5, 49.7)	58.4 (58.4, 58.5)
		>0.80	17.1 (17.1, 17.2)	26.2 (26.1, 26.3)	89.4 (89.3, 89.4)	62.8 (62.8, 62.9)	63.9 (63.7, 64.0)	
	FIB4	<0.80	21.4 (21.3, 21.4)	89.3 (89.3, 89.4)	29.1 (29.0, 29.1)	79.2 (79.8, 79.3)	47.4 (47.4, 47.5)	62.3 (62.2, 62.4)
		>2.30	16.3 (16.3, 16.4)	24.5 (24.5, 24.6)	89.6 (89.5, 89.6)	62.4 (62.3, 62.4)	62.8 (62.6, 62.9)	
Elastography group (n = 817)	VCTE	<6.0	29.6 (29. 6, 29.7)	89.0 (88.9, 89.1)	42.7 (42.7, 42.9)	84.6 (84.5, 84.7)	52.3 (52.2, 52.4)	54.9 (54.8, 54.9)
		>15.4	15.5 (15.5, 15.6)	23.9 (23.8, 24.0)	90.4 (90.3, 90.4)	62.7 (62.7, 62.8)	63.6 (63.5, 63.8)	
	Agile3+	<0.102	27.5 (27.4, 27.5)	88.1 (88.0, 88.1)	38.4 (38.3, 38.5)	82.0 (81.9, 82.1)	50.2 (50.1, 50.3)	56.1 (56.0, 56.2)
		>0.844	16.4 (16.4, 16.5)	25.1 (25.0, 25.2)	89.7 (89.7, 89.8)	62.9 (62.9, 63.0)	63.3 (63.1, 63.5)	
Specialized blood tests group (n = 545)	ELF	<8.6	23.2 (23.1, 23.2)	88.7 (88.6, 88.8)	33.1 (33.0, 33.3)	77.8 (77.6, 78.0)	52.7 (52.6, 52.8)	57.8 (57.7, 57.9)
		>10.4	19.1 (19.0, 19.2)	29.3 (29.2, 29.4)	89.5 (89.4, 89.6)	60.1 (60.0, 60.2)	70.1 (69.9, 70.3)	
	Fibrotest	<0.14	15.4 (15.3, 15.5)	89.9 (89.8, 90.0)	19.9 (19.8, 20.0)	70.2 (70.0, 70.4)	48.5 (48.4, 48.6)	72.5 (72.4, 72.6)
		>0.71	12.1 (12.1, 12.2)	15.2 (15.1, 15.3)	90.5 (90.4, 90.6)	56.0 (55.9, 56.1)	57.4 (57.2, 57.7)	

[a] Patients included in the interval defined by the diagnostic threshold; [b] Rate of patients in the interval between the two diagnostic thresholds.

Se: sensitivity; Spe: specificity; NPV: negative predictive value; PPV: positive predictive value

Table S5: Accuracy of non-invasive tests for the diagnosis of fibrotic MASH with optimized thresholds, as a function of the prevalence of fibrotic MASH

Group	Test	Threshold		Fibrotic MASH prevalence					
				5%	10%	20%	30%	40%	50%
Study population (n = 1,055)	FNI	<0.23	% Patients (NPV)	33.8 (99)	32.5 (97)	30.0 (93)	27.5 (89)	25.0 (84)	22.5 (78)
		0.23-0.80	% Patients	54.5	55.0	56.0	57.0	58.0	59.0
		>0.80	% Patients (PPV)	11.8 (11)	12.5 (21)	14.0 (37)	15.5 (50)	17.0 (61)	18.5 (70)
	FIB-4	<0.80	% Patients (NPV)	28.1 (98)	27.2 (96)	25.4 (91)	23.6 (86)	21.8 (80)	20.0 (73)
		0.80-2.30	% Patients	61.2	61.3	61.6	61.9	62.2	62.5
		>2.30	% Patients (PPV)	10.8 (12)	11.5 (22)	13.0 (38)	14.5 (52)	16.0 (63)	17.5 (71)
Elastography group (n = 817)	FAST	≤0.35	% Patients (NPV)	50.8 (99)	48.8 (97)	44.7 (94)	40.7 (91)	36.6 (86)	32.6 (81)
		0.35-0.67	% Patients	34.8	35.3	36.4	37.4	38.5	39.5
		≥0.67	% Patients (PPV)	14.4 (15)	15.9 (27)	18.9 (45)	21.9 (59)	24.9 (69)	27.9 (77)
	VCTE	<6.0	% Patients (NPV)	41.4 (99)	39.8 (97)	36.6 (94)	33.4 (90)	30.2 (85)	27.0 (80)
		6.0-15.4	% Patients	47.9	48.8	50.6	52.4	54.2	56.0
		>15.4	% Patients (PPV)	10.7 (11)	11.4 (21)	12.8 (38)	14.2 (51)	15.6 (62)	17.0 (71)
	Agile3+	<0.102	% Patients (NPV)	36.7 (98)	35.4 (97)	32.8 (93)	30.2 (88)	27.6 (83)	25.0 (76)
		0.102-0.844	% Patients	52.6	53.1	54.2	55.3	56.4	57.5
		>0.844	% Patients (PPV)	10.8 (12)	11.5 (22)	13.0 (38)	14.5 (52)	16.0 (63)	17.5 (71)
Specialized blood tests group (n = 545)	MACK-3	<0.135	% Patients (NPV)	38.2 (99)	36.6 (98)	33.2 (96)	29.9 (94)	26.5 (90)	23.2 (86)
		0.135-0.549	% Patients	44.8	45.0	45.5	45.9	46.4	46.8
		>0.549	% Patients (PPV)	17.0 (13)	18.4 (24)	21.3 (42)	24.2 (55)	27.1 (66)	30.1 (74)
	ELF	<8.6	% Patients (NPV)	31.9 (98)	30.8 (96)	28.6 (92)	26.4 (88)	24.2 (82)	22.0 (75)
		8.6-10.4	% Patients	57.2	57.3	57.6	57.9	58.2	58.5
		>10.4	% Patients (PPV)	11.0 (13)	11.9 (24)	13.8 (42)	15.7 (55)	17.6 (66)	19.5 (74)
	Fibrotest	<0.14	% Patients (NPV)	19.5 (97)	19.0 (95)	18.0 (89)	17.0 (82)	16.0 (75)	15.0 (67)
		0.14-0.71	% Patients	71.2	71.4	71.8	72.2	72.6	73.0
		>0.71	% Patients (PPV)	9.3 (8)	9.6 (16)	10.2 (29)	10.8 (42)	11.4 (53)	12.0 (63)

NPV: negative predictive value; PPV: positive predictive value

Table S6: Accuracy of non-invasive tests for the diagnosis of fibrotic MASH with optimized thresholds in the 747 patients with reliable VCTE

Group	Test	Threshold	Patients (%) [a]	Se (%)	Spe (%)	NPV (%)	PPV (%)	Grey zone (%) [b]
Elastography group (n = 747)	VCTE	<6.0	30.7	88	43	84	52	55.0
		>15.4	14.3	22	91	63	62	
	Agile3+	<0.102	22.1	88	39	83	50	57.0
		>0.844	14.9	22	90	63	59	

[a] Patients included in the interval defined by the diagnostic threshold
[b] Rate of patients in the interval between the two diagnostic thresholds
Se: sensitivity; Spe: specificity; NPV: negative predictive value; PPV: positive predictive value; VCTE: vibration controlled transient elastography

Table S7: Prevalence of fibrotic MASH in the diagnostic zones of non-invasive tests when they are used within the grey zone of FAST or MACK-3

Grey zone	Fibrosis test	Rule-out zone		Grey zone	Rule-in zone	
		Threshold	Fibrotic MASH (%)	Fibrotic MASH (%)	Threshold	Fibrotic MASH (%)
FAST	FNI	<0.23	33	52	>0.80	35
	FIB4	<0.80	34	51	>2.30	57
	VCTE	<6.0	28	55	>15.4	44
	Agile3+	<0.102	28	54	>0.844	67
	MACK-3	<0.135	20	48	>0.549	47
	ELF	<8.6	36	61	>10.4	64
	Fibrotest	<0.14	48	59	>0.71	59
MACK-3	FNI	<0.23	25	45	>0.80	42
	FIB4	<0.80	23	46	>2.30	50
	FAST	≤0.35	16	48	≥0.67	52
	VCTE	<6.0	19	48	>15.4	47
	Agile3+	<0.102	21	46	>0.844	52
	ELF	<8.6	28	52	>10.4	67
	Fibrotest	<0.14	40	52	>0.71	41

Table S8: FAST / Agile3+ and MACK-3 / ELF algorithms for the diagnosis of fibrotic MASH

	**MACK3 <0.135**	**MACK3 0.135-0.549**			**MACK3 >0.549**
		**ELF <8.6**	**ELF 8.6-10.4**	**ELF >10.4**	
**All (n)**	134	60	161	33	157
**No fibrotic NASH (n)**	118	43	78	11	46
**Fibrotic NASH (n)**	16	17	83	22	111

	**FAST ≤ 0.35**	**FAST 0.35-0.67**			**FAST ≥ 0.67**
		**Agile3+ <0.102**	**Agile 3+ 0.102-0.844**	**Agile 3+ > 0.844**	
**All (n)**	295	92	187	36	207
**No fibrotic NASH (n)**	253	66	86	12	62
**Fibrotic NASH (n)**	42	26	101	24	145

Table S9: Detailed histological characteristics of the patients as a function of the diagnostic intervals of the FAST / Agile3+ algorithm

Green boxes correspond to the patients well classified for fibrotic MASH, and grey boxes to those misclassified for fibrotic MASH.

Fibrosis stage	Rule out interval (n=387)			Grey zone (n=187)			Rule in interval (n=243)		
	No MASH	MASH & MAS = 3	MASH & MAS ≥4	No MASH	MASH & MAS = 3	MASH & MAS ≥4	No MASH	MASH & MAS = 3	MASH & MAS ≥4
0	85	18	27	9	1	6	3	0	2
1	46	26	58	14	9	18	6	1	21
2	20	19	51	5	8	57	4	6	46
3	12	7	17	5	8	36	7	4	87
4	1	0	0	2	1	8	11	9	36

MASH: metabolic associated-dysfunction steatohepatitis; MAS: MASLD activity score

Table S10: Detailed histological characteristics of the patients as a function of the diagnostic interval of the MACK-3 / ELF algorithm

Green boxes correspond to the patients well classified for fibrotic MASH, and grey boxes to those misclassified for fibrotic MASH.

Fibrosis stage	Rule out interval (n=194)			Grey zone (n=161)			Rule in interval (n=190)		
	No MASH	MASH & MAS = 3	MASH & MAS ≥4	No MASH	MASH & MAS = 3	MASH & MAS ≥4	No MASH	MASH & MAS = 3	MASH & MAS ≥4
0	35	0	6	8	0	6	1	0	1
1	41	15	15	7	8	12	3	4	11
2	17	19	26	8	9	47	2	8	35
3	5	6	6	9	10	32	8	8	69
4	2	0	1	1	0	4	7	4	29

MASH: metabolic associated-dysfunction steatohepatitis; MAS: MASLD activity score

Fig. S1: Observed probability of fibrotic MASH as a function of tests results

The LOWESS curve by weighted least squares (in red) was used to determine the average trend of relationships between the tests results and the presence of fibrotic MASH (Y axis on the right).

Yellow bars represent the number of patients included in each test interval (Y axis on the left).

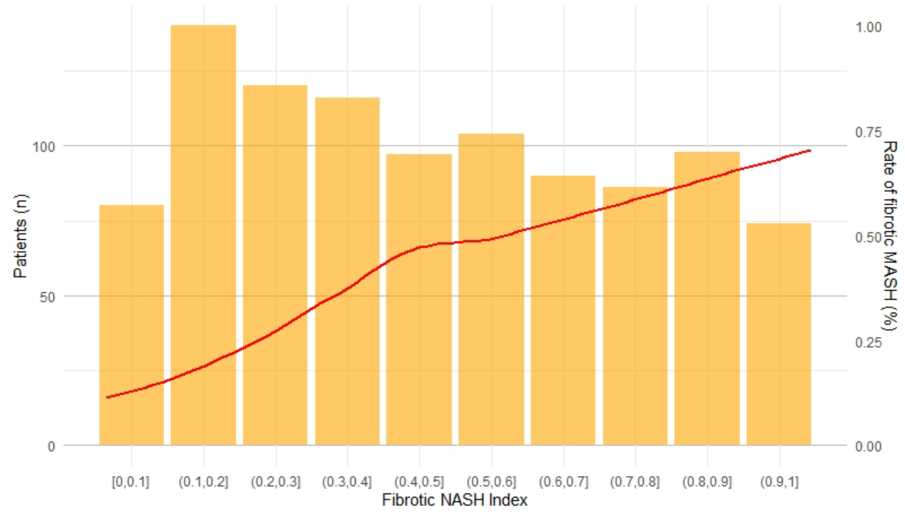

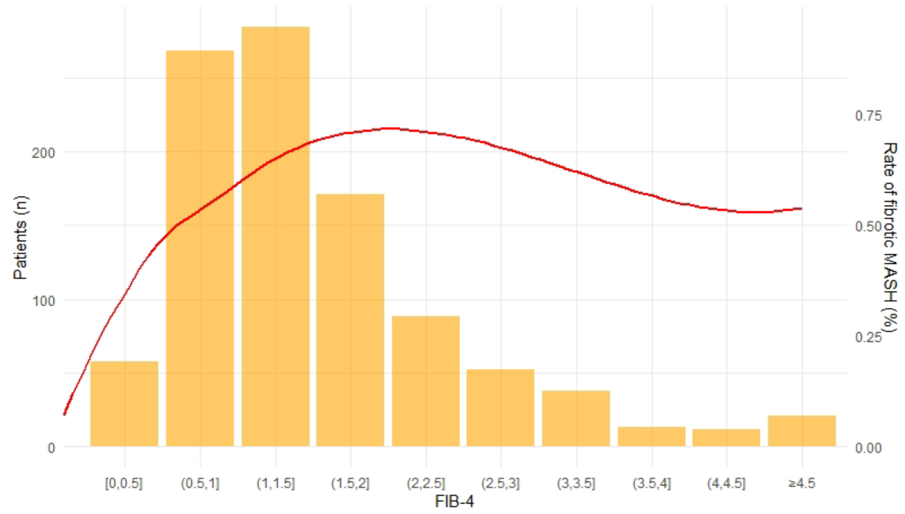

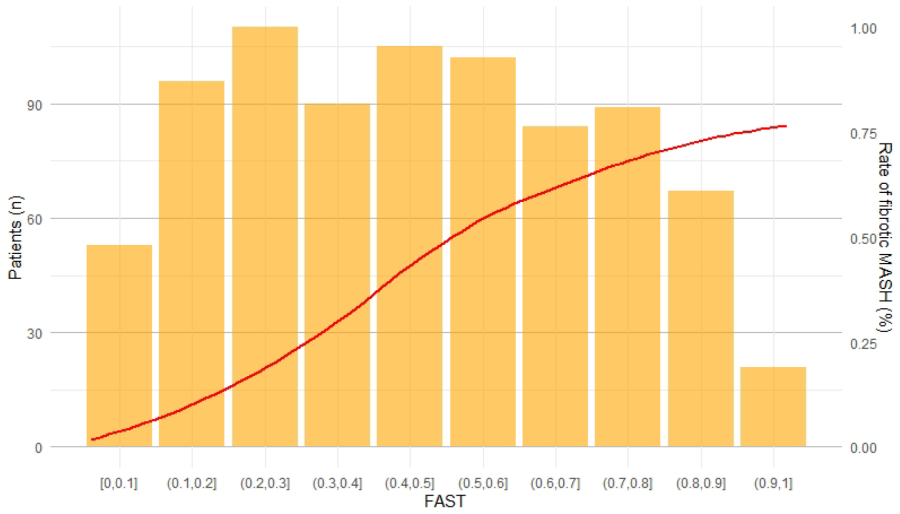

Patients (n)
0
30
60
90
Rate of fibrotic MASH (%)
0.00
0.25
0.50
0.75
1.00
[0,0.1]
(0.1,0.2]
(0.2,0.3]
(0.3,0.4]
(0.4,0.5]
(0.5,0.6]
(0.6,0.7]
(0.7,0.8]
(0.8,0.9]
(0.9,1]
FAST

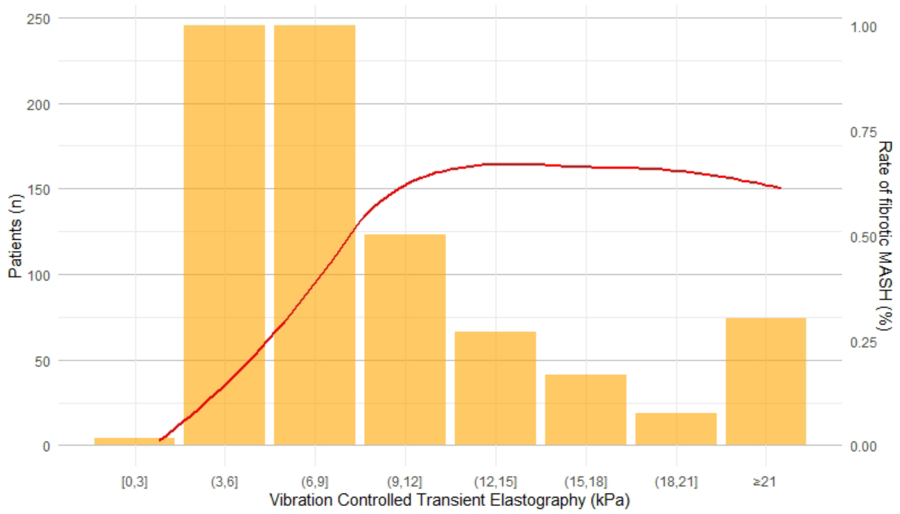

Patients (n)
0
50
100
150
200
250
Rate of fibrotic MASH (%)
0.00
0.25
0.50
0.75
1.00
[0,3]
(3,6]
(6,9]
(9,12]
(12,15]
(15,18]
(18,21]
≥21
Vibration Controlled Transient Elastography (kPa)

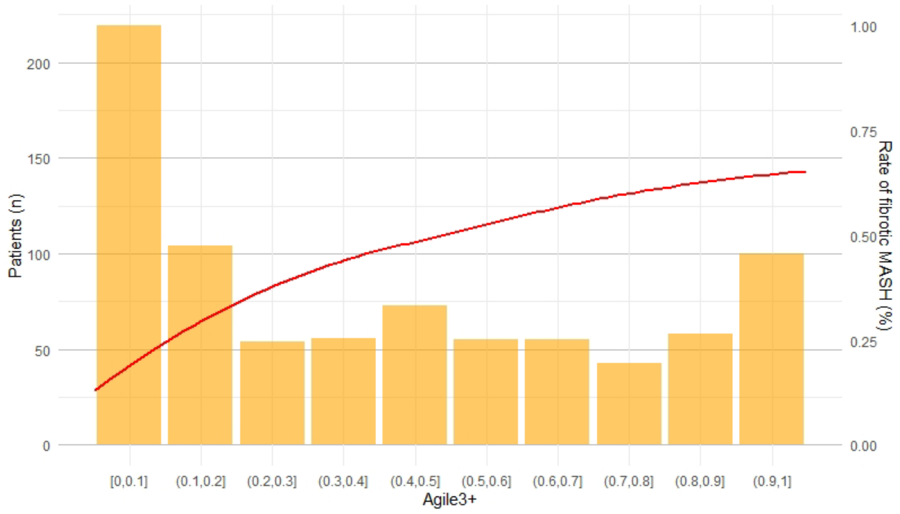

Patients (n)
0
50
100
150
200
Rate of fibrotic MASH (%)
0.00
0.25
0.50
0.75
1.00
[0,0.1]
(0.1,0.2]
(0.2,0.3]
(0.3,0.4]
(0.4,0.5]
(0.5,0.6]
(0.6,0.7]
(0.7,0.8]
(0.8,0.9]
(0.9,1]
Agile3+

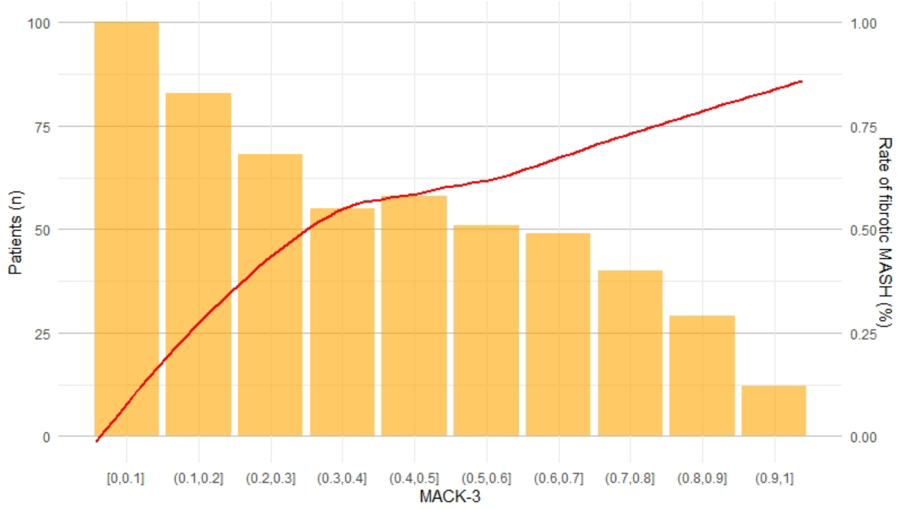

Patients (n)
Rate of fibrotic MASH (%)
100
75
50
25
0
1.00
0.75
0.50
0.25
0.00
[0,0.1]
(0.1,0.2]
(0.2,0.3]
(0.3,0.4]
(0.4,0.5]
(0.5,0.6]
(0.6,0.7]
(0.7,0.8]
(0.8,0.9]
(0.9,1]
MACK-3

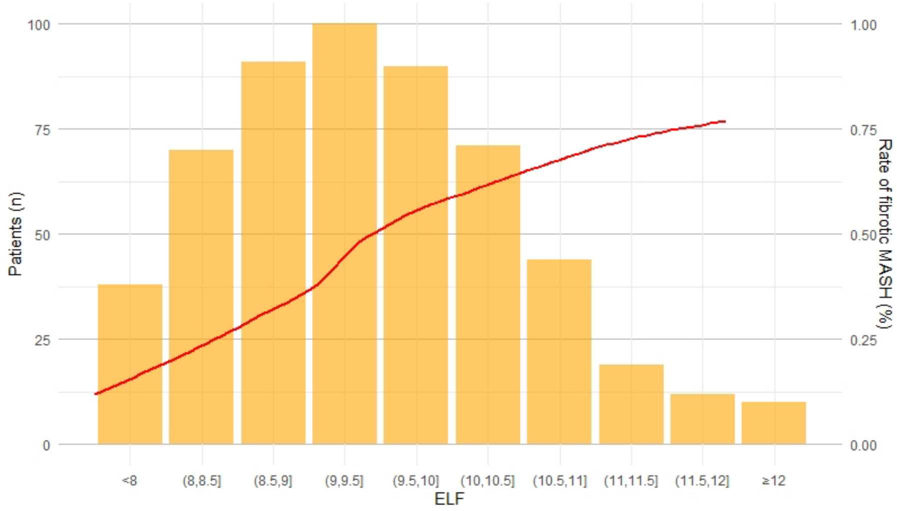

Patients (n)
Rate of fibrotic MASH (%)
100
75
50
25
0
1.00
0.75
0.50
0.25
0.00
<8
(8,8.5]
(8.5,9]
(9,9.5]
(9.5,10]
(10,10.5]
(10.5,11]
(11,11.5]
(11.5,12]
≥12
ELF

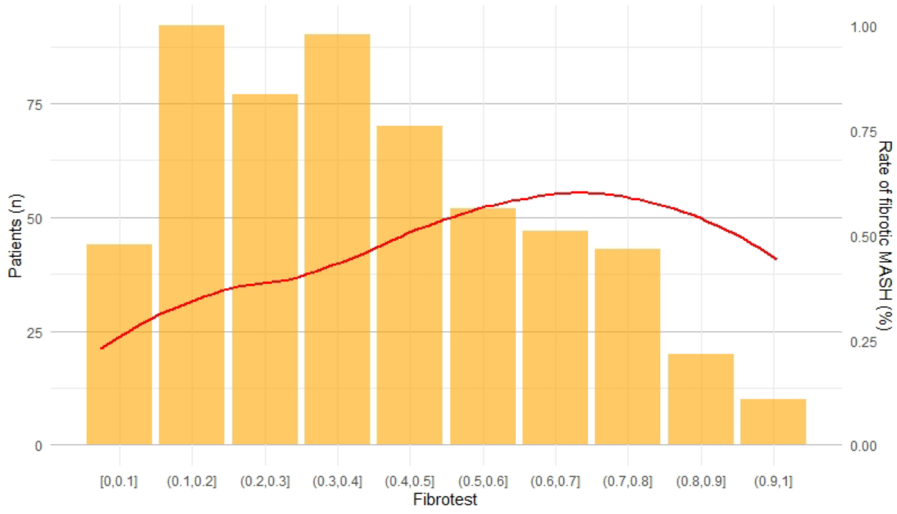

Patients (n)
Rate of fibrotic MASH (%)
75
50
25
0
1.00
0.75
0.50
0.25
0.00
[0,0.1]
(0.1,0.2]
(0.2,0.3]
(0.3,0.4]
(0.4,0.5]
(0.5,0.6]
(0.6,0.7]
(0.7,0.8]
(0.8,0.9]
(0.9,1]
Fibrotest

Fig. S2: Results (mean with 95% confidence interval) of non-invasive tests between patients with or without fibrotic MASH, in each of the three diagnostic zones of FAST

FNI:

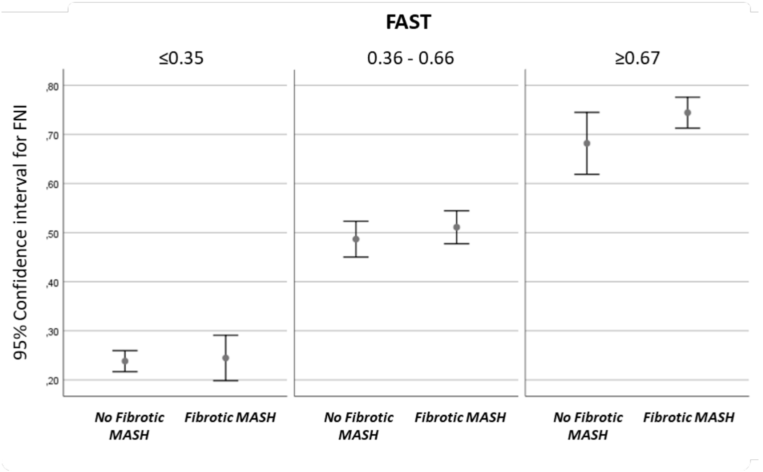

FIB4:

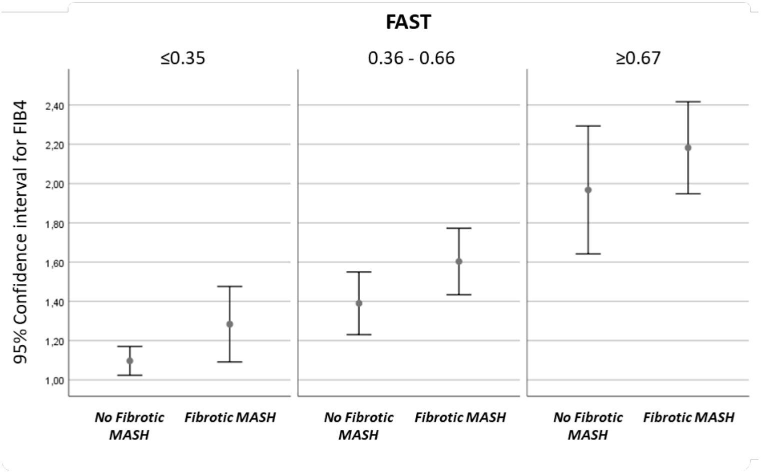

VCTE:

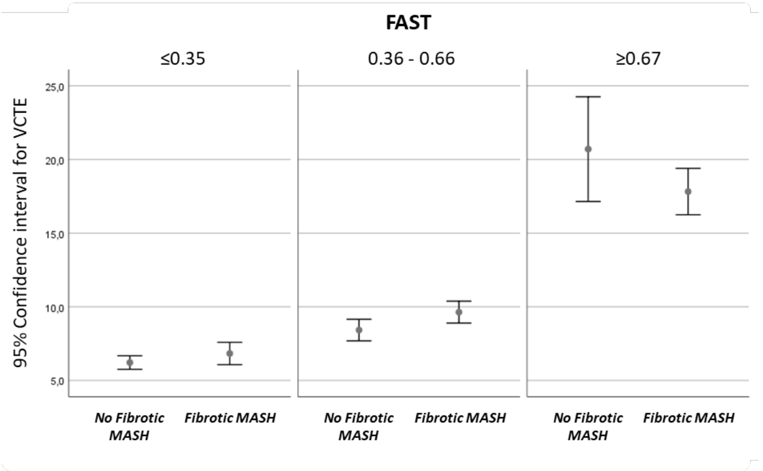

Agile3+:

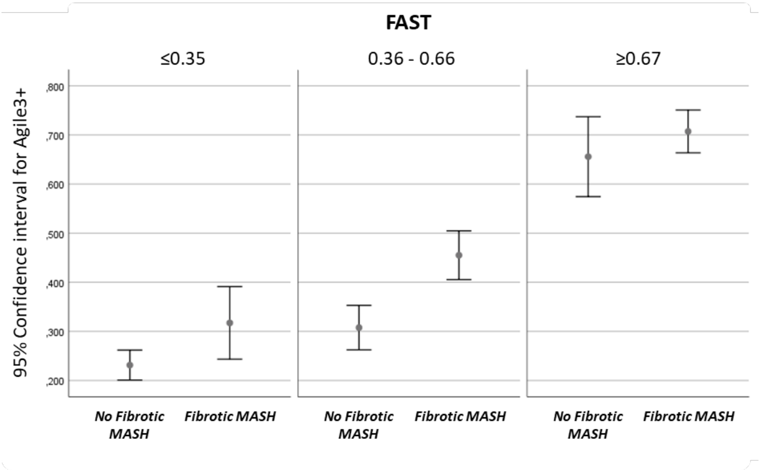

MACK-3:

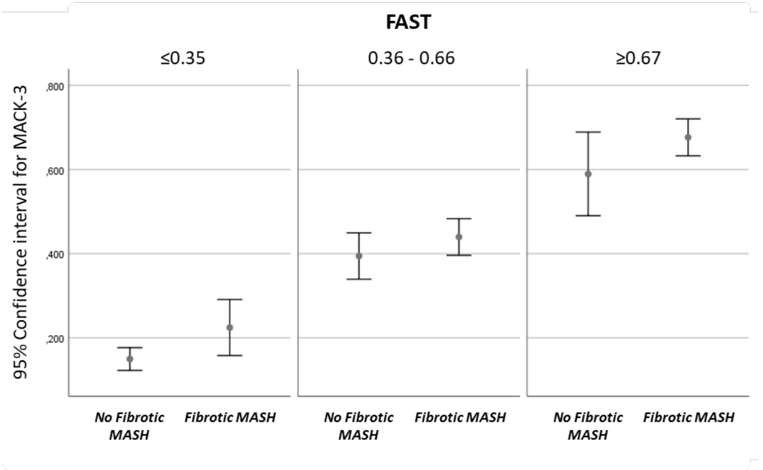

ELF:

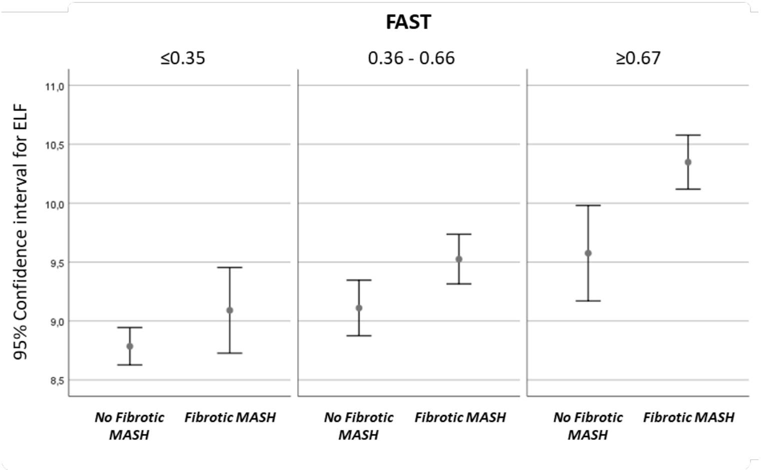

Fibrotest:

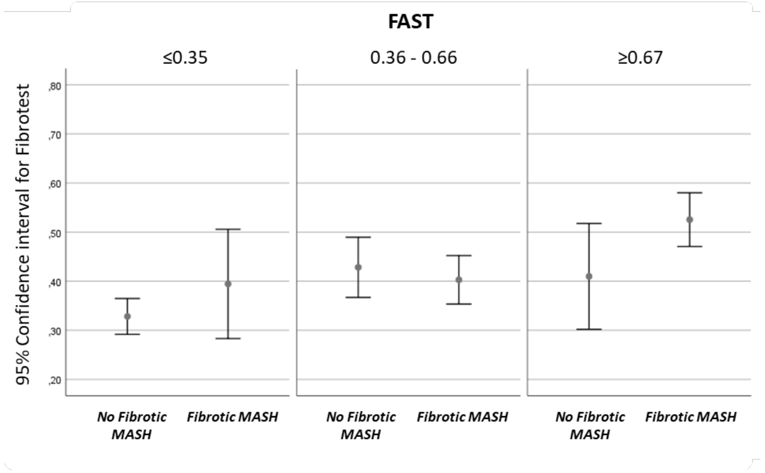

Fig. S3: Results for the FAST / Agile3+ algorithm in the 747 patients with reliable VCTE

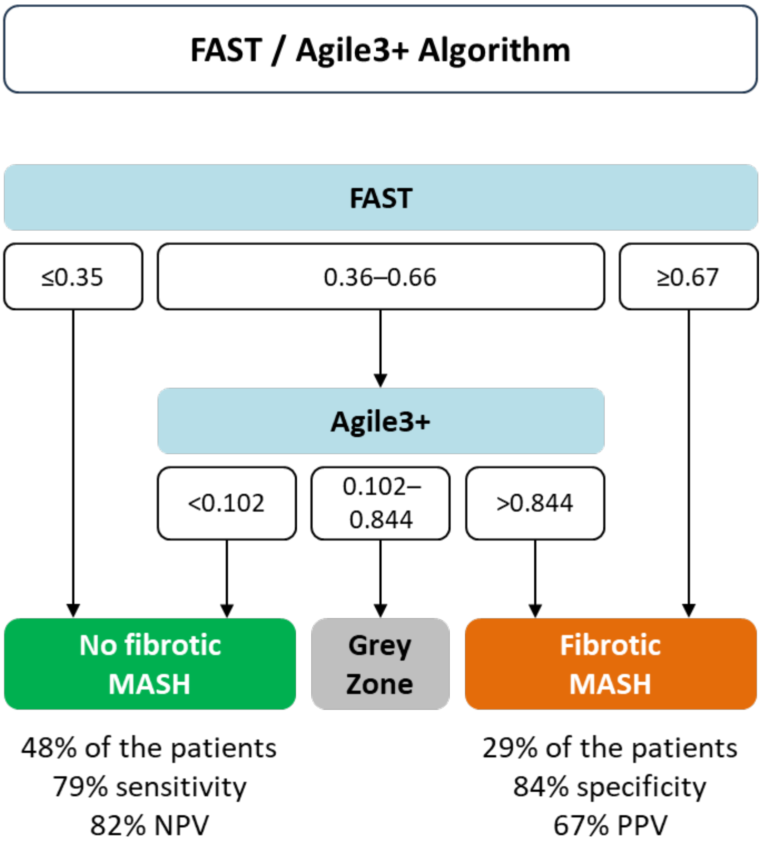

Fig. S4: Results (mean with 95% confidence interval) of non-invasive tests between patients with or without fibrotic MASH, in each of the three diagnostic zones of MACK-3

FNI:

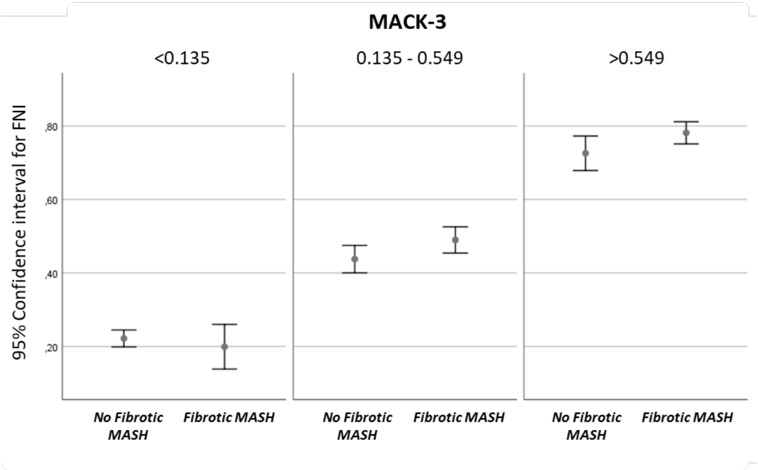

FIB4:

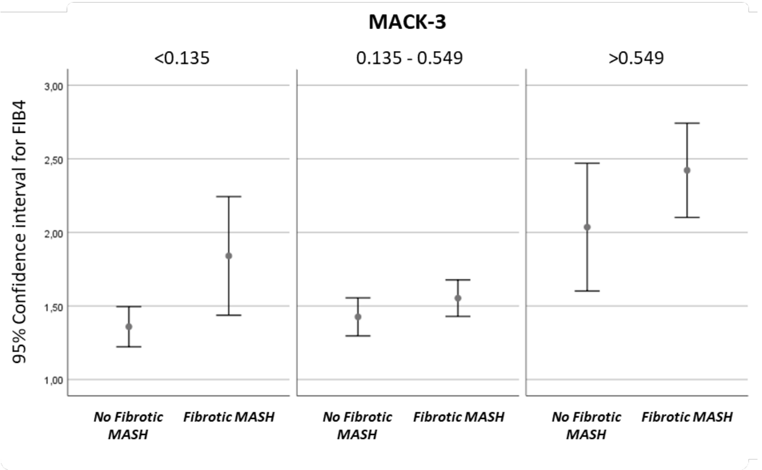

FAST:

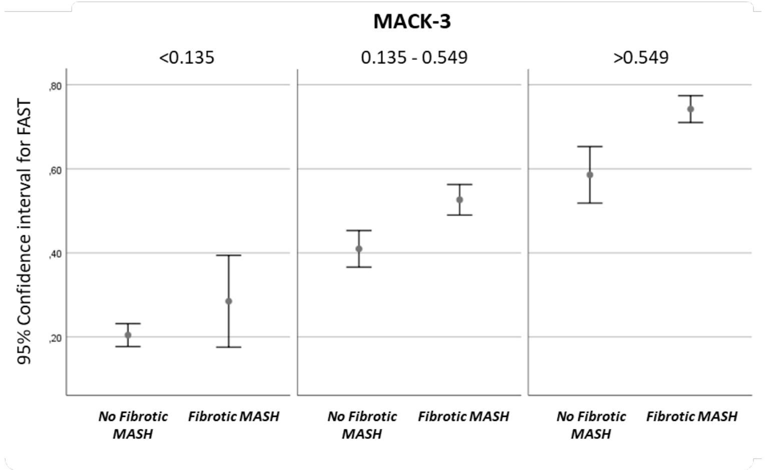

VCTE:

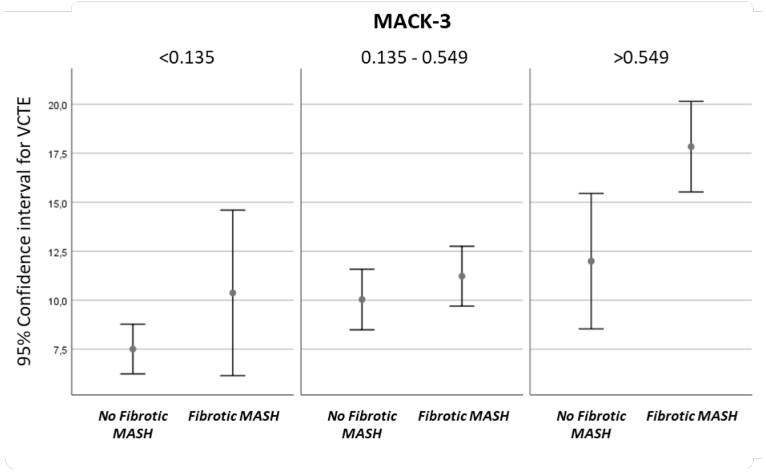

Agile3+:

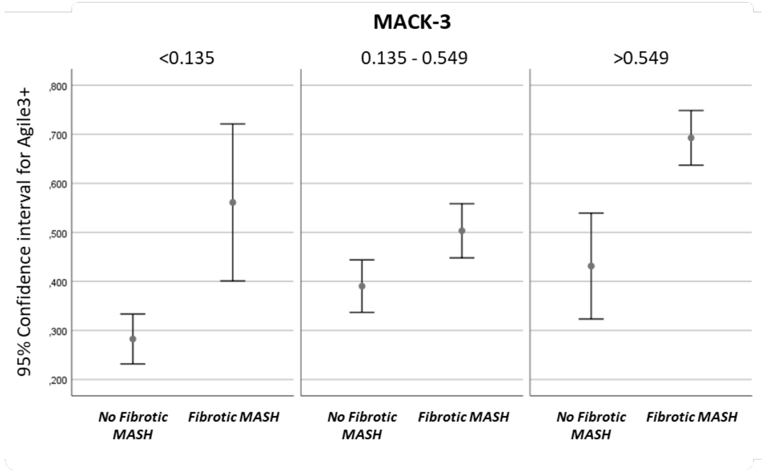

ELF:

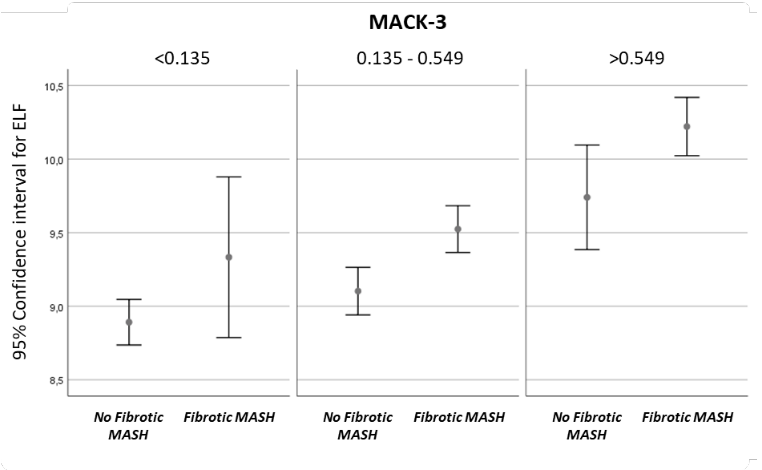

Fibrotest:

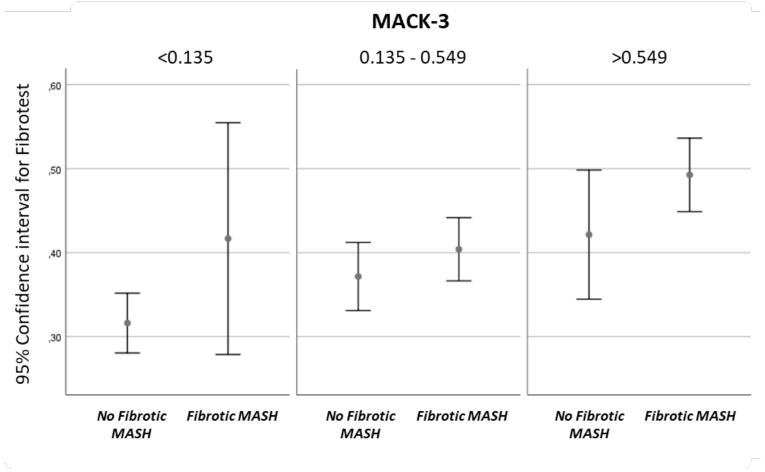

Supplement: Multimedia component 1 [file mmc1.pdf]
