# Supplementary material for: Non-invasive tests for fibrotic MASH for reducing screen failure in therapeutic trials
Source: JHEP Rep. 2025 Feb 4;7(4):101351. doi: 10.1016/j.jhepr.2025.101351 (PMC11985113; doi:10.1016/j.jhepr.2025.101351)

Research article

JHEP Reports

Non-invasive tests for fibrotic MASH for reducing screen failure in therapeutic trials

Jeanne Fichez[1,2], Thomas Mouillot[3], Luisa Vonghia[4,5], Charlotte Costentin[6,7], Clémence Moreau[2,8], Marine Roux[2], Adèle Delamarre[9], Sven Francque[4,5], Ming-Hua Zheng[10], Jérôme Boursier[1,2,*]

JHEP Reports **2025**. vol. 7 | 1–10

Check for updates

Background & Aims: Therapeutic trials in metabolic dysfunction-associated steatohepatitis (MASH) are hampered by a high 70–80% screen failure rate mostly because of the absence of fibrotic MASH on baseline liver biopsies, underscoring the need for better selection of candidates. We compared the performance of eight non-invasive tests, designed or not for the diagnosis of fibrotic MASH.

Methods: A total of 1,005 patients with histologically proven MASLD were included in five tertiary care centers. Three non-invasive tests developed for fibrotic MASH were evaluated: the simple blood test Fibrotic NASH Index (FNI), the specialized blood test MACK-3, and the elastography-based test FAST. Five non-invasive tests recommended for advanced fibrosis were evaluated as well: the simple blood test FIB-4, the specialized blood tests FibroTest and Enhanced Liver Fibrosis test (ELF^{TM}), and the elastography-based tests FibroScan and Agile3+. Fibrotic MASH was defined as MASH with MASLD activity score ≥4 and fibrosis score F ≥2.

Results: Among simple blood tests (n = 1,005), FNI had a significantly higher area under the receiver operating characteristic (AUROC) for fibrotic MASH than FIB-4 (0.709 [0.677–0.741] *vs.* 0.662 [0.628–0.695], p = 0.019). Among elastography-based tests (n = 817), FAST had a significantly higher AUROC (0.774 [0.743–0.806]) than FibroScan (0.728 [0.694–0.763], p = 0.013) and Agile3+ (0.708 [0.672–0.744], p = 0.004). Among specialized blood tests (n = 545), MACK-3 had a significantly higher AUROC (0.772 [0.734–0.811]) than FibroTest (0.615 [0.568–0.663], p <0.001) and ELF (0.700 [0.656–0.744], p = 0.028). Sequential combination (FAST then Agile3+; MACK-3 then ELF) identified a subset including one-third of patients in whom the false-positive rate was only 30%.

Conclusions: Sequential combinations using first-line tests designed for fibrotic MASH improves the identification of candidates for MASH therapeutic trials.

© 2025 The Authors. Published by Elsevier B.V. on behalf of European Association for the Study of the Liver (EASL). This is an open access article under the CC BY license (http://creativecommons.org/licenses/by/4.0/).

Introduction

Metabolic dysfunction-associated steatotic liver disease (MASLD) describes the accumulation of lipids in the liver in a context of obesity and insulin resistance, without excessive alcohol consumption and other causes of chronic liver diseases.[1] Metabolic dysfunction-associated steatohepatitis (MASH) is characterized by the association of liver steatosis with lobular inflammation and hepatocyte ballooning; it is the aggressive form of MASLD and promotes fibrosis accumulation. Fibrosis is the main prognostic factor in MASLD, with the risk of liver-related complications becoming significant from the F2 fibrosis stage onwards and increasing exponentially across the F3 and F4 stages.

In parallel with the growing pandemic of obesity, MASLD has become the leading cause of chronic liver disease and MASH is now among the leading causes of cirrhosis, hepatocellular carcinoma, and liver transplantation worldwide.[2–4] Blood tests and elastography devices have been developed for the non-invasive assessment of liver fibrosis and the identification of asymptomatic patients with advanced MASLD requiring specialized management. These non-invasive tests (NITs) were initially developed and evaluated for the non-invasive diagnosis of advanced fibrosis, that is, F3–4 fibrosis according to the MASH Clinical Research Network (MASH CRN) semiquantitative histological scoring system.[5] Evidence on their accuracies has reached the level of meta-analyses,[6–8] and scientific societies now agree on their use in clinical practice for the diagnosis of advanced fibrosis.[9–13]

Drug development for MASH has expanded considerably over the past decade, with the first drug recently approved by the FDA in March 2024[14] and numerous phase II and III trials underway. The target population for inclusion in MASH therapeutic trials is usually defined as having biopsy-proven fibrotic MASH, that is, MASH with a MASLD activity score (MAS) ≥4

* Corresponding author. Address: Service d'Hépato-Gastroentérologie et Oncologie Digestive, Centre Hospitalier Universitaire, 4 rue Larrey, 49933 Angers cedex 09, France; Tel.: +33 2 41 35 34 10; fax: +33 2 41 35 41 19.
E-mail address: JeBoursier@chu-angers.fr (J. Boursier).
https://doi.org/10.1016/j.jhepr.2025.101351

and a fibrosis stage F ≥2. The absence of such histologically confirmed fibrotic MASH on the screening liver biopsy is one of the main causes of the high screen failure rate of around 70–80% currently encountered in these trials. There is thus a need to improve trial candidate selection. In contrast to their ability to assess the risk of advanced fibrosis, accuracy of the currently available and recommended NITs is lower to assess significant fibrosis (*i.e.* F2) and no test has shown acceptable accuracy to assess MASH as such. In this context, a new generation of NITs has been recently developed specifically for the diagnosis of fibrotic MASH. These include the simple blood test Fibrotic NASH Index (FNI),[15] the specialized blood test MACK-3,[16] and the elastography-based test FAST.[17] These tests are aimed at improving the screening of candidates for clinical trials and, once new drugs come to market, allowing the identification of the patients who will need them.

However, it remains currently unclear as to whether these new tests specifically designed for fibrotic MASH outperform the NITs currently available in clinical practice in this setting of trial screening. Therefore, in the work presented here, we aimed to evaluate and directly compare the performance of eight NITs, designed or not for the diagnosis of fibrotic MASH.

Patients and methods

Patients

The study population was obtained by pooling the data from five cohorts of patients with MASLD from tertiary care centers in France (Angers, Bordeaux, and Grenoble), Belgium (Antwerp), and China (Wenzhou). The Angers cohort enrolled patients from 2010 to 2022, and the other cohorts from 2015 to 2020. The included patients underwent liver biopsy as part of their MASLD investigation after exclusion of concomitant steatosis-inducing drugs (such as corticosteroids, tamoxifen, amiodarone, or methotrexate), excessive alcohol consumption (>210 g/week in men or >140 g/week in women), chronic hepatitis B or C infection, and histological evidence of any other concomitant chronic liver disease.[1] For a patient to be included in the present study, Fibrosis-4 (FIB-4) and FNI had to be available, as well as the three elastography-based tests FibroScan, FAST, and Agile 3+ (elastography group) or the three specialized blood tests MACK-3, ELF™, and FibroTest (specialized blood tests group). Patients were excluded if they had a liver biopsy with length <10 mm, or a history of liver-related complications. All patients were recruited from hepatology clinics, and none of the liver biopsies were performed during bariatric surgery. All five cohorts obtained approval from Ethics Committees: CPP Ouest II Angers (CB2010-01) for Angers; CPP Sud-Ouest et Outre Mer III for Bordeaux; ARS Rhone Alpes (AC-2014-2094) for Grenoble; Ethisch Comite UZA (15/21/227) for Antwerp; and Ethics Committee of the First Affiliated Hospital of Wenzhou Medical University (2016-246) for Wenzhou. Approval for the cohorts covered the work carried out here. All patients gave written informed consent before inclusion.

Histology

Pathological examinations were performed in each center by a same senior expert specialized in hepatopathology and blinded to patient data. We and others have shown excellent interobserver reproducibility for liver fibrosis evaluation when performed by expert pathologists.[5,16,18] Liver fibrosis stage (F) was evaluated according to the MASH CRN scoring system, that is, F0: no fibrosis; F1: perisinusoidal or portal/periportal fibrosis; F2: perisinusoidal and portal/periportal fibrosis; F3: bridging fibrosis; and F4: cirrhosis.[5] MASH was defined as a grade of 1 or higher in each component of steatosis, lobular inflammation, and hepatocellular ballooning. The MAS (ranging from 0 to 8) corresponded to the sum of the steatosis, lobular inflammation, and ballooning grades.[5] Fibrotic MASH, the primary diagnostic target of the study, was defined as the presence of MASH with MAS ≥4 and F ≥2. Advanced fibrosis was defined as F3 + F4 fibrosis stages (F3–4).

Blood-based fibrosis tests

Fasting blood samples were taken on the day or within a week of the liver biopsy. Five blood tests were calculated according to published or patented formulas (Table S1). Two of them were specifically developed for the diagnosis of fibrotic MASH: the simple blood test FNI, and the specialized blood test MACK-3. The thresholds used with these tests to rule-out or rule-in fibrotic MASH were 0.10/0.33 for FNI,[15] and 0.135/0.549 for MACK-3.[16] Three blood tests were developed for the diagnosis of advanced fibrosis: the simple blood test FIB-4, and the specialized blood tests ELF and FibroTest. The thresholds used with these blood tests to rule-out or rule-in advanced fibrosis were 1.30/2.67 for FIB-4, 0.32/0.48 for FibroTest, and 7.7/9.8 for ELF.[19]

Liver stiffness measurement and elastography-based tests

Liver stiffness measurement was performed using vibration-controlled transient elastography (VCTE) with the FibroScan device (Echosens, Paris, France), and by experienced operators blinded to patient and histological data. VCTE examinations were performed within 1 month of the liver biopsy, with patients in fasting condition, and according to the manufacturer's recommendations. We note here that VCTE alone is not 'dedicated' as are the other tests evaluated in the present work but its effectiveness in diagnosing advanced fibrosis is well established and recommended by international guidelines.[9–13] Therefore, it was grouped among the advanced fibrosis tests in our study. The thresholds used with VCTE to rule-out or rule-in advanced fibrosis were 8.0/12.0 kPa.[6,20] VCTE examinations with a result ≥8 kPa and an IQR/median ratio ≤0.30 were considered unreliable.[21,22] Two elastography-based tests combining VCTE results with other data were calculated according to published formulas (Table S1). These were FAST, which was specifically developed for the diagnosis of fibrotic MASH (rule-out and rule-in thresholds: 0.35/0.67), and Agile3+, which was specifically developed for the diagnosis of advanced fibrosis (rule-out and rule-in thresholds: 0.451/0.679).[17,23]

Statistical analysis

Continuous variables were expressed as medians, with first and third quartiles, and compared using the Mann–Whitney test. Categorical variables were expressed as percentages and compared using the Fisher test. Diagnostic accuracy was evaluated using the area under the receiver operating curve (AUROC) and compared with the Delong test. When necessary,

diagnostic thresholds were recalculated to reach 90% sensitivity (rule-out threshold) and 90% specificity (rule-in threshold), and further validated internally in 1,000 bootstrap samples, each one corresponding to a random selection of two-thirds of the study population.

Three patient groups were considered for the analyses: the study population, the elastography group, and the specialized blood tests group (Table S2). FNI and FIB-4 were available in all the 1,005 patients included in the study population. This enabled the direct comparison of two simple blood tests designed for different diagnostic targets (FNI to fibrotic MASH, and FIB-4 to advanced fibrosis). VCTE results were available in 817 patients (elastography group), enabling the direct comparison of VCTE and the two elastography-based tests designed for different diagnostic targets (FAST to fibrotic MASH and Agile3+ to advanced fibrosis). In this group, we also evaluated the added value of elastography-based tests compared to the simple blood tests FNI and FIB-4. Finally, MACK-3, ELF, and FibroTest were available in 545 patients (specialized blood tests group), which allowed the direct comparison of three specialized blood tests designed for different diagnostic targets (MACK-3 to fibrotic MASH, ELF and FibroTest to advanced fibrosis), as well as the evaluation of the added value of specialized *vs.* simple blood tests.

The *p* values were adjusted for multiple comparisons using the Holm-Bonferroni method. Statistical analyses were performed using R version 4.0.5 (R Foundation for Statistical Computing, Vienna, Austria).

Results

Patients

The characteristics of the 1,005 patients included in the study are summarized in Table 1. Median age was 56.7 years, 62.7% were male, median BMI was 31.0 kg/m^2, and 49.0% of the patients had type 2 diabetes. Biopsy length was 10–19 mm in 16.8% of the patients and ≥20 mm in 83.2%. The prevalence of fibrotic MASH and advanced fibrosis were, respectively, 41.8% and 31.9%. Characteristics of the elastography group and the specialized blood tests group were very close to those of the whole study population.

AUROC analysis

Table 2 provides a summary of the AUROCS for the diagnosis of fibrotic MASH and advanced fibrosis. In the whole population and considering the diagnosis of fibrotic MASH, the thereto-designed FNI had a significantly higher AUROC than FIB-4 did, and conversely, considering advanced fibrosis, the thereto-designed FIB-4 had a significantly higher AUROC than FNI.

In the elastography group and considering fibrotic MASH, the thereto-designed FAST had a significantly higher AUROC than Agile3+ and VCTE, and conversely, considering advanced fibrosis, the thereto-designed Agile3+ and VCTE had significantly higher AUROCs than FAST. The elastography-based tests performed better than the simple blood tests for their

Table 1. Patients' characteristics.

	Study population (N = 1,005)	Elastography group (n = 817)	Specialized blood tests group (n = 545)
Age (years)	56.7 (45.7–64.8)	56.0 (44.4–65.0)	59.2 (50.0–66.4)
Male sex (n, %)	630 (62.7)	516 (63.2)	342 (52.8)
BMI (kg/m^2)	31.0 (27.5–35.5)	30.6 (27.2–35.5)	32.0 (29.0–36.9)
Type 2 diabetes (n, %)	492 (49.0)	390 (47.7)	288 (52.8)
Biopsy length (mm)	28 (22–35)	28 (21–35)	29 (23–35)
MASLD activity score	4 (3–5)	4 (3–5)	4 (3–5)
MASH (n, %)	719 (71.5)	587 (71.8)	391 (71.7)
Fibrotic MASH (n, %)	420 (41.8)	338 (41.4)	249 (45.7)
Fibrosis stage (n, %):			
- 0	165 (16.4)	151 (18.5)	57 (10.5)
- 1	242 (24.1)	199 (24.4)	116 (21.3)
- 2	277 (27.6)	216 (26.4)	171 (31.4)
- 3	239 (23.8)	183 (22.4)	153 (28.1)
- 4	82 (8.2)	68 (8.3)	48 (8.8)
AST (IU/L)	37 (27–52)	36 (27–52)	39 (29–52)
ALT (IU/L)	53 (36–79)	52 (35–79)	53 (35–78)
GGT (IU/L)	63 (38–114)	60 (38–107)	71 (40–130)
Bilirubin (µmol/L)	10 (8–15)	11 (8–15)	10 (7–14)
Albumin (g/L)	43.4 (40.6–46.0)	44 (41–47)	43 (40–45)
Platelets (G/L)	223 (184–263)	225 (185–266)	213 (173–256)
Prothrombin time (%)	98 (91–106)	99 (92–106)	97 (89–104)
FNI	0.44 (0.23–0.71)	0.42 (0.21–0.69)	0.45 (0.26–0.73)
FIB-4	1.28 (0.86–1.89)	1.26 (0.84–1.85)	1.44 (1.01–2.09)
MACK-3	0.284 (0.113–0.990)	0.286 (0.112–0.563)	0.337 (0.136–0.585)
ELF	9.4 (8.7–10.1)	9.4 (8.6–10.1)	9.4 (8.6–10.1)
FibroTest	0.37 (0.20–0.56)	0.38 (0.21–0.57)	0.37 (0.20–0.56)
VCTE (kPa)	8.1 (5.7–12.1)	7.8 (5.6–11.8)	8.4 (5.9–12.6)
FAST	0.46 (0.25–0.67)	0.46 (0.25–0.67)	0.49 (0.26–0.67)
Agile3+	0.368 (0.100–0.701)	0.355 (0.092–0.700)	0.442 (0.137–0.757)

ALT, alanine aminotransferase; AST, aspartate aminotransferase; BMI, body mass index; ELF, Enhanced Liver Fibrosis test; FIB-4, Fibrosis-4; FNI, Fibrotic NASH Index; GGT, gamma-glutamyl transferase; MASH, metabolic dysfunction-associated steatohepatitis; MASLD, metabolic dysfunction-associated steatotic liver disease; VCTE, vibration-controlled transient elastography.

Table 2. AUROC for the diagnosis of fibrotic MASH and the diagnosis of advanced fibrosis.

	Global population (N = 1,055)	
Diagnostic target	Fibrotic MASH	Advanced fibrosis
**Global population (N = 1,055)**		
FNI	0.709 (0.677–0.741)	0.699 (0.665–0.734)
FIB-4	0.662 (0.628–0.695)	0.785 (0.755–0.815)
p value (comparison):		
FNI *vs.* FIB-4	0.019	<0.001
**Elastography group (n = 817)**		
FNI	0.709 (0.673–0.744)	0.705 (0.666–0.743)
FIB-4	0.668 (0.631–0.705)	0.792 (0.760–0.825)
FAST	0.774 (0.743–0.806)	0.768 (0.734–0.803)
VCTE	0.728 (0.694–0.763)	0.819 (0.788–0.849)
Agile3+	0.708 (0.672–0.744)	0.855 (0.828–0.882)
p values (comparison):		
FNI *vs.* FIB-4	0.442	0.002
FNI *vs.* FAST	<0.001	<0.001
FNI *vs.* VCTE	0.743	<0.001
FNI *vs.* Agile 3+	0.971	<0.001
FIB-4 *vs.* FAST	<0.001	0.732
FIB-4 *vs.* VCTE	0.027	0.757
FIB-4 *vs.* Agile3+	0.027	<0.001
FAST *vs.* VCTE	0.013	0.004
FAST *vs.* Agile3+	0.004	<0.001
Agile3+ *vs.* VCTE	0.467	0.012
**Specialized blood tests group (n = 545)**		
FNI	0.722 (0.680–0.765)	0.722 (0.677–0.768)
FIB-4	0.637 (0.591–0.684)	0.771 (0.731–0.811)
MACK-3	0.772 (0.734–0.811)	0.749 (0.707–0.791)
FibroTest	0.615 (0.568–0.663)	0.753 (0.711–0.794)
ELF	0.700 (0.656–0.744)	0.851 (0.819–0.883)
p values (comparison)		
FNI *vs.* FIB-4	0.020	0.543
FNI *vs.* MACK-3	0.005	0.552
FNI *vs.* FibroTest	0.003	1.000
FNI *vs.* ELF	1.000	<0.001
FIB-4 *vs.* MACK-3	<0.001	0.817
FIB-4 *vs.* FibroTest	1.000	1.000
FIB-4 *vs.* ELF	0.017	<0.001
MACK-3 *vs.* FibroTest	<0.001	0.894
MACK-3 *vs.* ELF	0.028	<0.001
FibroTest *vs.* ELF	0.005	<0.001

ELF test, Enhanced Liver Fibrosis test; FIB-4, Fibrosis-4; FNI, Fibrotic NASH Index; MASH, metabolic dysfunction-associated steatohepatitis; VCTE, vibration-controlled transient elastography.
AUROC were compared using the Delong test.

designed diagnostic targets: FAST had a significantly higher AUROC than FNI for fibrotic MASH and Agile3+ a significantly higher AUROC than FIB-4 for advanced fibrosis. VCTE was unreliable in 70 patients (8.6%). Restricting the analysis to the subgroup of patients with reliable VCTE did not change the study results (Table S3).

Comparable results were found in the specialized blood tests group. For the diagnosis of fibrotic MASH, the thereto-designed MACK-3 had a significantly higher AUROC compared with the advanced-fibrosis-designed ELF or FibroTest. Conversely, ELF had a significantly higher AUROC compared to MACK-3 for the diagnosis of advanced fibrosis. Specialized blood tests outperformed simple blood tests for their designed diagnostic targets: MACK-3 had a significantly higher AUROC than FNI for fibrotic MASH, and similarly ELF had a significantly higher AUROC than FIB-4 for advanced fibrosis.

Thus, NITs performed better for the diagnostic target they were designed for, and specialized tests (either blood or elastography-based) provided greater accuracy than simple blood tests did.

Diagnostic thresholds for fibrotic MASH

Fig. S1 shows the probability of fibrotic MASH as a function of the tests' results. The specialized tests designed for fibrotic MASH (FNI, FAST, and MACK-3) showed the best profile: the probability of fibrotic MASH progressively increased with their results to finally reach 75% (FNI and FAST) and even 85% (MACK-3) for high test values. Among the NITs intended for advanced fibrosis, Agile3+ and ELF showed also a progressive increase in the probability of fibrotic MASH as a function of their result. The probability of fibrotic MASH reached 75% in high ELF values, but this concerned less patients than with MACK-3. For the other NITs intended for advanced fibrosis (FIB-4, VCTE, and FibroTest), the probability of fibrotic MASH rapidly plateaued and did not exceed 60% in high test values.

Published thresholds

Table 3 shows the diagnostic accuracy for fibrotic MASH of NITs used with their published thresholds. FAST and MACK-3 ruled out or ruled in their designed diagnostic target with

Table 3. Accuracy of non-invasive tests for the diagnosis of fibrotic MASH with their published thresholds.

Test	Threshold	Patients (%)*	Se (%)	Spe (%)	NPV (%)	PPV (%)	Gray zone (%)†
**Study population (N = 1,055)**							
FNI	≤0.10	8.0	97	11	82	44	29.6
	≥0.33	62.4	81	51	79	55	
FIB-4	<1.30	51.2	63	61	70	54	37.8
	>2.67	11.0	17	93	61	64	
**Elastography group (n = 817)**							
FAST	≤0.35	36.1	88	53	86	57	38.6
	≥0.67	25.3	43	87	68	70	
VCTE	<8.0	50.8	72	67	77	60	24.7
	>12.0	24.5	37	85	66	63	
Agile3+	<0.451	57.5	61	71	72	59	17.3
	>0.679	25.2	39	84	66	64	
**Specialized blood test group (n = 545)**							
MACK-3	<0.135	24.6	94	40	88	57	46.6
	>0.549	28.8	45	84	64	71	
ELF	<7.7	2.9	100	5	94	47	62.6
	>9.8	34.5	49	77	64	64	
FibroTest	<0.32	43.7	65	51	64	53	21.1
	>0.48	35.2	44	72	61	57	

ELF test, Enhanced Liver Fibrosis test; FIB-4, Fibrosis-4; FNI, Fibrotic NASH index; NPV, negative predictive value; PPV, positive predictive value; Se, sensitivity; Spe, specificity; VCTE, vibration-controlled transient elastography.
*Patients included in the interval defined by the diagnostic threshold.
†Rate of patients in the interval between the two diagnostic thresholds.

sensitivities and specificities around 90%. FNI excluded fibrotic MASH with excellent 97% sensitivity, but only 8% of the patients were included in the rule-out zone. Moreover, specificity with the rule-in threshold was very low at 51%. FIB-4, Agile3+, and FibroTest provided low 61–65% sensitivity with their rule-out thresholds, and 72–93% specificity with their rule-in thresholds. VCTE showed a profile similar to those of the tests above designed for advanced fibrosis, with 72% sensitivity for results <8 kPa, and 85% specificity for results >12 kPa. An ELF result <7.7 provided perfect 100% sensitivity, but this concerned <3% of the patients. ELF >9.8 provided a specificity for fibrotic MASH (77%) similar to those of the other tests designed for advanced fibrosis.

These results confirmed that the published diagnostic thresholds of FAST and MACK-3 designed for fibrotic MASH are well-designed for the diagnosis of this condition, but not those of FNI. Conversely, and as expected, the published diagnostic thresholds of tests developed for advanced fibrosis are not well suited to the diagnosis of fibrotic MASH.

Optimized thresholds

The diagnostic cut-offs for FNI, FIB-4, VCTE, Agile3+, ELF, and FibroTest were recalculated with the aim of attaining 90% sensitivity for the rule-out threshold and 90% specificity for the rule-in threshold (Table 4). The diagnostic accuracies of the optimized thresholds were internally validated in 1,000 bootstrap samples (Table S4). These optimized thresholds doubled the size of the gray zone to 55–73% of the patients; this was higher than with the published thresholds for FAST and MACK-3 (Fig. 1). The negative and positive predictive values with these thresholds as a function of the prevalence of fibrotic MASH are

Table 4. Accuracy of non-invasive tests for the diagnosis of fibrotic MASH with optimized thresholds.

Test	Threshold	Patients (%)*	Se (%)	Spe (%)	NPV (%)	PPV (%)	Gray zone (%)†
**Study population (N = 1,055)**							
FNI	<0.23	24.5	90	35	83	50	58.4
	>0.80	17.1	26	89	63	64	
FIB-4	<0.80	21.4	89	29	79	47	62.3
	>2.30	16.3	25	90	62	63	
**Elastography group (n = 817)**							
VCTE	<6.0	29.6	89	43	85	52	54.8
	>15.4	15.5	24	90	63	64	
Agile3+	<0.102	27.4	88	38	82	50	56.2
	>0.844	16.4	25	90	63	63	
**Specialized blood test group (n = 545)**							
ELF	<8.6	23.1	89	33	78	53	57.8
	>10.4	19.1	29	90	60	70	
FibroTest	<0.14	15.4	90	20	70	49	72.5
	>0.71	12.1	15	91	56	58	

The optimized thresholds were calculated to obtain 90% sensitivity with the rule-out cut-off and 90% specificity with the rule-in cut-off.
ELF test, Enhanced Liver Fibrosis test; FIB-4, Fibrosis-4; FNI, Fibrotic NASH Index; NPV, negative predictive value; PPV, positive predictive value; Se, sensitivity; Spe, specificity; VCTE, vibration-controlled transient elastography.
*Patients included in the interval defined by the diagnostic threshold.
†Rate of patients in the interval between the two diagnostic thresholds.

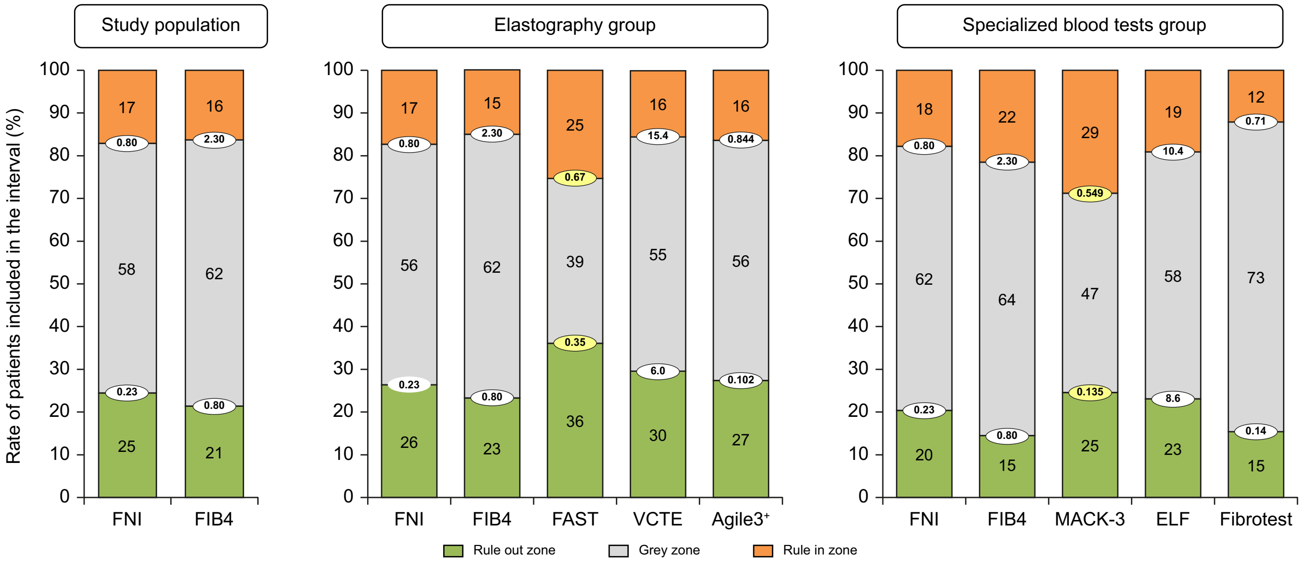

Fig. 1. Rate of patients included in the diagnostic intervals of the non-invasive tests using the diagnostic thresholds for fibrotic MASH. The diagnostic thresholds for FAST and MACK-3 (in yellow) were those previously published as they were confirmed to provide the targeted sensitivity and specificity (see Table 3). The diagnostic thresholds for the other tests were recalculated and optimized in the study population to reach 90% sensitivity (rule-out threshold) and 90% specificity (rule-in threshold). ELF test, Enhanced liver fibrosis test; FIB-4, Fibrosis-4; FNI, Fibrotic NASH Index; VCTE, vibration-controlled transient elastography.

presented in Table S5. In the elastography group, restricting the analysis to the subgroup of patients with reliable VCTE did not change the study results (Table S6). Finally, the specialized tests FAST and MACK-3 designed for fibrotic MASH provided the best compromise for this diagnostic target, as they combined high sensitivities, high specificities, and a small gray zone.

Tests combination for the diagnosis of fibrotic MASH

We further evaluated whether the sequential use of NITs could help identify more patients with fibrotic MASH within the gray zones of FAST and MACK-3.

FAST/Agile3+ algorithm

In the gray zone of FAST, Agile3+ was the test that best discriminated patients with fibrotic MASH from those without (Fig. S2). Moreover, the rule-in zone of Agile3+ provided the highest prevalence of fibrotic MASH within the gray zone of FAST (67%, Table S7). The sequential combination of these two elastography-based tests (FAST/Agile3+ algorithm, Fig. 2 and Table S8) diagnosed 30% of the patients as having fibrotic MASH. In this group, the rate of false positives was only 30% (70% positive predictive value), which is much lower than the current 70–80% screening failure rate in MASH therapeutic trials. Sensitivity for fibrotic MASH was 80% with the FAST/

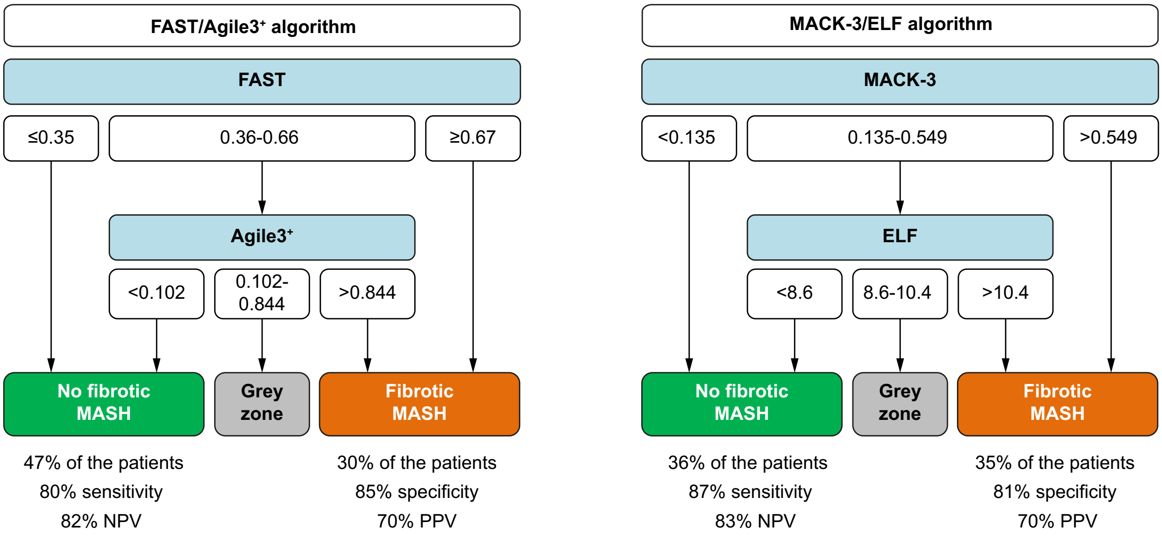

Fig. 2. FAST/Agile3+ and MACK-3/ELF algorithms for the diagnosis of fibrotic MASH. ELF, Enhanced Liver Fibrosis test; MASH, metabolic dysfunction-associated steatohepatitis; NPV, negative predictive value; PPV, positive predictive value.

Agile3+ algorithm. Restricting the analysis to the subgroup of patients with reliable VCTE did not change the study results (Fig. S3).

MACK-3/ELF algorithm

In the gray zone of MACK-3, FAST and ELF were the tests that best discriminated patients with fibrotic MASH from those without (Fig. S4). The rule-in zone of ELF provided the highest prevalence of fibrotic MASH within the gray zone of MACK-3 (67% *vs.* 52% for FAST; Table S7). The sequential combination of the two specialized blood tests, MACK-3 then ELF, (MACK-3/ELF algorithm, Fig. 2 and Table S8), diagnosed 35% of patients as having fibrotic MASH. In this group, the rate of false positives was only 30%. Sensitivity for fibrotic MASH was 87% with the MACK-3/ELF algorithm.

Analysis of false positives

Fig. 3 and Table S9 show the detailed histological characteristics of the patients as a function of the diagnostic intervals of the FAST/Agile3+ algorithm. Among the 74 patients misclassified for fibrotic MASH in the rule-in interval, 19 (26%) had MASH with fibrosis F2–4 but MAS = 3 and 18 (24%) had advanced fibrosis without the presence of MASH. Finally, only 15% of the patients in the rule-in interval of the FAST/Agile3+ algorithm had mild liver disease (F0–1 fibrosis, or F2 fibrosis without MASH; Fig. 3).

Among the 57 patients misclassified for fibrotic MASH in the rule-in interval of the MACK-3/ELF algorithm, 20 (35%) had MASH with fibrosis F2–4 but MAS = 3 and 15 (26%) had advanced fibrosis without the presence of MASH (Table S10). Finally, only 12% of the patients in the rule-in interval of the MACK-3/ELF algorithm had mild liver disease (F0–1 fibrosis, or F2 fibrosis without MASH; Fig. 3).

Discussion

To our knowledge, our work is the first direct comparison of the NITs currently recommended for the assessment of advanced fibrosis in patients with MASLD[9–13] to those newly developed for the diagnosis of fibrotic MASH. We found that NITs specifically developed to fibrotic MASH were more accurate for this diagnostic target than those developed for advanced liver fibrosis. We also confirmed that specialized tests (MACK-3, FAST) perform better than simple blood tests (FNI) in fibrotic MASH, a finding echoing earlier comparisons of specialized and simple tests for advanced fibrosis.[22] Finally, we developed two algorithms that sequentially combine specialized tests to improve the non-invasive diagnosis of fibrotic MASH: the elastography-based FAST/Agile3+ algorithm, and the blood-based MACK-3/ELF algorithm. Both identify a subset enriched in patients with fibrotic MASH where the rate of false positives is only 30%. These algorithms will considerably improve the identification of candidates for the therapeutic trials in MASH. The strengths of our work are: the very large number of included patients (N = 1,005); the multicentric design with the entire spectrum of MASLD lesions represented in the study population; the direct comparison of several non-invasive test types (simple, specialized) using different modalities (biology, elastography) and aimed at different diagnostic targets (fibrotic MASH, advanced liver fibrosis); the good quality of liver biopsies used as reference; and histological readings performed by specialized and expert pathologists in each center. Finally, our AUROCs for advanced liver fibrosis in this study were very close to those previously published in large meta-analyses,[6–8] reinforcing the relevance of our study data and results.

The NITs initially developed for advanced fibrosis (FIB-4, Agile3+, ELF, FibroTest) showed poor accuracy when evaluated for the diagnosis of fibrotic MASH, with AUROCs around 0.60–0.70. Thus, as expected, the published thresholds for these tests were not well suited for fibrotic MASH. Attempting to refine those thresholds specifically for fibrotic MASH led to an expansion of the gray zone to 50–70% of patients. In contrast, tests designed for fibrotic MASH provided the highest AUROCs for it, significantly outperforming their counterparts designed for advanced fibrosis. The published diagnostic thresholds of FAST and MACK-3 were confirmed to provide not

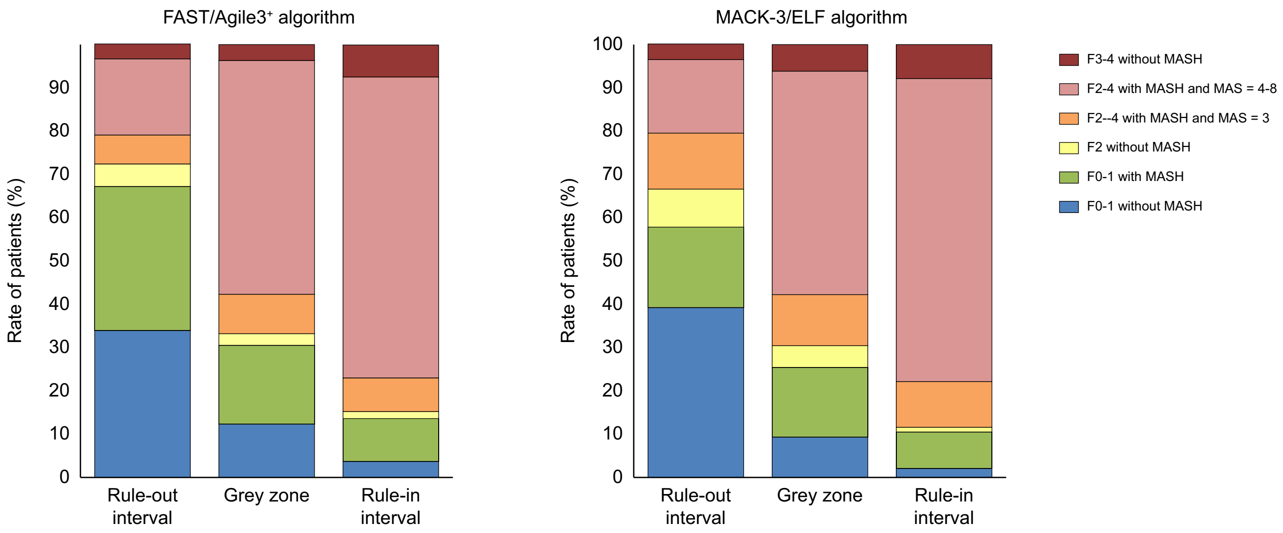

Fig. 3. Prevalence of MASLD lesions as a function of the three diagnostic intervals of FAST/Agile3+ and MACK-3/ELF algorithms. ELF, Enhanced liver fibrosis test; MAS, MASLD activity score; MASLD, metabolic dysfunction-associated steatotic liver disease.

only high sensitivity and specificity, but also the smallest gray zone. We emphasize that these better results observed for FAST and MACK-3 surpassed an optimism bias. Indeed, we used the published thresholds for those two tests whereas we specifically refined the thresholds for the other tests in the current study population. The published thresholds for FNI (≤0.10 and ≥0.33) were not optimal in our study. Its thresholds were initially calculated in a population of patients who underwent bariatric surgery,[15] which corresponds to a particular context. That suboptimal performance led us to refine the FNI thresholds to <0.23 and >0.80. These numbers should be more pertinent for patients managed in the setting of hepatology clinics. Our analysis focused on AUROCs for advanced fibrosis showed that FIB-4 performed better than FNI (simple blood tests), VCTE and Agile3+ performed better than FAST (elastography-based tests), and ELF was more accurate than MACK-3 (specialized blood tests). Our results thus demonstrate that NITs, which address a specific question by construction, should be preferably used for the diagnostic target for which they were developed.

An interesting finding in our study is that Agile3+ and ELF, although inferior for fibrotic MASH as a first-line test, are of added value to further stratify the risk of fibrotic MASH in the gray zones of FAST and MACK-3 respectively. Indeed, because almost all the patients with advanced fibrosis have fibrotic MASH, high values for the tests developed for advanced fibrosis are associated with good specificity for fibrotic MASH. We have previously shown the value of the concept of test complementarity for the diagnosis of cirrhosis,[24] and our results here suggest that this concept also applies for fibrotic MASH. Other innovative solutions are being developed to improve the non-invasive diagnosis of fibrotic MASH. MRI-proton density fat fraction (MRI-PDFF) is the non-invasive gold standard for liver steatosis assessment,[9] and magnetic resonance elastography is the best method for the non-invasive diagnosis of liver fibrosis.[25] The MRI-aspartate aminotransferase (MAST) score is an MRI-based approach that takes advantage of these two technologies to diagnose fibrotic MASH. In the development study, MAST showed a very good AUROC at 0.93 in the validation set[26] but two subsequent independent studies reported less convincing AUROCs at 0.72 and 0.79.[27,28] A recently developed circulating proteomic signature provided very good accuracy and an AUROC at 0.85 for fibrotic MASH in the validation set of the development study.[29] As this signature requires a specific proteomic platform to be measured, it would probably be positioned as a second-line test for fibrotic MASH. Perilipin 2 (PLIN2) is a protein associated with the metabolism of intracellular lipid droplets.[30] A recent study suggested the measurement of PLIN2 by flow cytometry in peripheral blood CD14+CD16-monocytes as an accurate biomarker for MASH, providing >90% accuracy.[31] In that study, combining PLIN2 with diabetes, waist circumference, triglycerides, and serum transaminase produced an AUROC of 98% for the non-invasive diagnosis of MASH, but accuracy for fibrotic MASH was not evaluated. Going forward, further work is now mandatory to determine how these new non-invasive solutions can interact with already available tests to improve the diagnosis of fibrotic MASH in clinical practice.

By delaying study recruitment and inducing important additional costs, the high 70–80% screen failure rate in therapeutic trials considerably hampers drug development in MASH. NITs represent a highly attractive solution for identifying a subset of patients enriched with fibrotic MASH. The LITMUS consortium recently proposed that the screen failure rate in MASH therapeutic trials should ideally not exceed 33%.[32] In line with this proposal, the two algorithms we propose here, FAST/Agile3+ and MACK-3/ELF, represent readily available and easy-to-implement solutions that will facilitate inclusions. Both algorithms offer a similar risk stratification profiles: around one-third of the patients were diagnosed by the algorithm to have fibrotic MASH, and the rate of false positives in those groups did not exceed 30%, well below the 70–80% mainly biopsy-driven screen failure rate observed in MASH therapeutic trials. FAST/Agile3+ has the practical advantage of being a single-step algorithm: both FAST and Agile3+ rely on VCTE results and can therefore be calculated simultaneously. However, FibroScan devices remain insufficiently available with regards to the very large population of patients with MASLD to evaluate. Many ultrasound devices now incorporate liver steatosis and stiffness measurements, opening the possibility of developing new ultrasonographic tests inspired by the FAST concept to disseminate the diagnosis of fibrotic MASH in clinical practice. In comparison, MACK-3/ELF has the advantage of being a fully blood-based algorithm. Because laboratory tests are ubiquitous and more easily accessible, primary care physicians, diabetologists, obesity specialists and cardiologists can easily adopt the MACK-3/ELF algorithm for the identification of fibrotic MASH cases among their patients.

The main limitations of our work are the selection of patients with a subsequent increased prevalence of fibrotic MASH, the histological reading by a single pathologist at each institution, and missing data for specialized blood tests which did not allow direct comparison of all NITs in a single cohort with large sample size. Liver biopsy is no longer performed in the absence of evidence of significant liver disease, which mechanically results in an enrichment of significant fibrosis in the cohorts including patients with biopsy-proven MASLD. This is the case in the ongoing prospective cohorts led by large multicentric international consortia that are investigating the non-invasive diagnosis of liver lesions. Indeed, the prevalence of significant fibrosis F ≥2 was reported to be 49% in the European LITMUS cohort and 69% in the US NIMBLE cohort.[32,33] Both the LITMUS and the NIMBLE use liver biopsy with central reading as a reference to assess the accuracy of NITs. The AUROCs they reported for ELF, VCTE, and MACK-3 are close to those in our study, suggesting that our histological reference is relevant.[32,33]

In conclusion, NITs specifically designed for fibrotic MASH are more accurate for this diagnostic target than tests currently recommended and initially developed for advanced fibrosis. The use of first-line tests designed for fibrotic MASH and, even better, sequential non-invasive test combinations will foster the identification of people with fibrotic MASH in need of treatment, and their inclusion in MASH therapeutic trials.

Affiliations

[1]Hepato-Gastroenterology and Digestive Oncology Department, Angers University Hospital, Angers, France; [2]HIFIH Laboratory, SFR ICAT 4208, Angers University, Angers, France; [3]Hepato-Gastroenterology and Digestive Oncology Department, Dijon University Hospital, Dijon, France; [4]Department of Gastroenterology and Hepatology, Antwerp University Hospital, Antwerp, Belgium; [5]Laboratory of Experimental Medicine and Pediatrics, University of Antwerp, Antwerp, Belgium; [6]Grenoble Alpes University/Hepato-Gastroenterology and Digestive Oncology Department, Grenoble Alpes University Hospital, Grenoble, France; [7]Grenoble Institute for Advanced Biosciences, Research Center UGA/Inserm U 1209/CNRS 5309, Grenoble Alpes University, Grenoble, France; [8]Department of Methodology and Biostatistics, Angers University Hospital, Angers, France; [9]Hepatology Unit, Haut Leveque Hospital, Bordeaux University Hospital, Bordeaux, France; [10]MAFLD Research Center, Department of Hepatology, The First Affiliated Hospital of Wenzhou Medical University, Wenzhou, China

Abbreviations

ALT, alanine aminotransferase; AST, aspartate aminotransferase; AUROC, area under the receiver operating curve; ELF test, Enhanced Liver Fibrosis test; FIB-4, Fibrosis-4; FNI, Fibrotic NASH Index; Gamma-GT, gamma-glutamyl transferase; MAS, MASLD Activity Score; MASH, metabolic dysfunction-associated steatohepatitis; MASLD, metabolic dysfunction-associated steatotic liver disease; MAST, MRI-aspartate aminotransferase; MRI-PDFF, MRI-proton density fat fraction; NITs, non-invasive tests; NPV, negative predictive value; PLIN2, perilipin 2; PPV, positive predictive value; Se, sensitivity; Spe, specificity; VCTE, vibration-controlled transient elastography.

Financial support

Grant received from Angers University Hospital.

Conflicts of interest

JB reports consulting activities with Echosens. All other authors declare no conflicts of interest that pertain to this work.

Please refer to the accompanying ICMJE disclosure forms for further details.

Authors' contributions

Study design: TM, JB. Data acquisition: LV, CC, AD, SF, MHZ, JB. Analysis: JF, CM, MR, JB. Drafting and critical revision: all authors.

Data availability statement

The data that support the findings of this study are available from the corresponding author upon request.

Acknowledgements

We thank M. de Saint Loup, S. Girre, P. Celea, and A. Klingberg for collecting data and building the database.

Supplementary data

Supplementary data to this article can be found online at https://doi.org/10.1016/j.jhepr.2025.101351.

References

Author names in bold designate shared co-first authorship

[1] Rinella ME, Lazarus JV, Ratziu V, et al. A multisociety Delphi consensus statement on new fatty liver disease nomenclature. J Hepatol 2023;79:1542–1556.

[2] Younossi ZM, Golabi P, Paik JM, et al. The global epidemiology of nonalcoholic fatty liver disease (NAFLD) and nonalcoholic steatohepatitis (NASH): a systematic review. Hepatology 2023;77:1335–1347.

[3] Wong VW, Ekstedt M, Wong GL, et al. Changing epidemiology, global trends and implications for outcomes of NAFLD. J Hepatol 2023;79:842–852.

[4] Huang DQ, Singal AG, Kono Y, et al. Changing global epidemiology of liver cancer from 2010 to 2019: NASH is the fastest growing cause of liver cancer. Cell Metab 2022;34:969. 77.e962.

[5] Kleiner DE, Brunt EM, Van Natta M, et al. Design and validation of a histological scoring system for nonalcoholic fatty liver disease. Hepatology 2005;41:1313–1321.

[6] Mozes FE, Lee JA, Selvaraj EA, et al. Diagnostic accuracy of non-invasive tests for advanced fibrosis in patients with NAFLD: an individual patient data meta-analysis. Gut 2022;71:1006–1019.

[7] Vali Y, Lee J, Boursier J, et al. Enhanced liver fibrosis test for the non-invasive diagnosis of fibrosis in patients with NAFLD: a systematic review and meta-analysis. J Hepatol 2020;73:252–262.

[8] Vali Y, Lee J, Boursier J, et al. FibroTest for evaluating fibrosis in non-alcoholic fatty liver disease patients: a systematic review and meta-analysis. J Clin Med 2021;10:2415.

[9] European Association for the Study of the Liver. EASL Clinical Practice Guidelines on non-invasive tests for evaluation of liver disease severity and prognosis - 2021 update. J Hepatol 2021;75:659–689.

[10] Rinella ME, Neuschwander-Tetri BA, Siddiqui MS, et al. AASLD Practice Guidance on the clinical assessment and management of nonalcoholic fatty liver disease. Hepatology 2023;77:1797–1835.

[11] Kanwal F, Shubrook JH, Adams LA, et al. Clinical care pathway for the risk stratification and management of patients with nonalcoholic fatty liver disease. Gastroenterology 2021;161:1657–1669.

[12] Cusi K, Isaacs S, Barb D, et al. American association of clinical endocrinology clinical practice guideline for the diagnosis and management of nonalcoholic fatty liver disease in primary care and endocrinology clinical settings: Co-sponsored by the American association for the study of liver diseases (AASLD). Endocr Pract 2022;28:528–562.

[13] European Association for the Study of the Liver. EASL-EASD-EASO Clinical Practice Guidelines on the management of metabolic dysfunction-associated steatotic liver disease (MASLD). J Hepatol 2024;81:492–542.

[14] Harrison SA, Bedossa P, Guy CD, et al. A phase 3, randomized, controlled trial of resmetirom in NASH with liver fibrosis. N Engl J Med 2024;390:497–509.

[15] Tavaglione F, Jamialahmadi O, De Vincentis A, et al. Development and validation of a score for fibrotic nonalcoholic steatohepatitis. Clin Gastroenterol Hepatol 2023;21:1523. 32.e1521.

[16] Boursier J, Anty R, Vonghia L, et al. Screening for therapeutic trials and treatment indication in clinical practice: MACK-3, a new blood test for the diagnosis of fibrotic NASH. Aliment Pharmacol Ther 2018;47:1387–1396.

[17] Newsome PN, Sasso M, Deeks JJ, et al. FibroScan-AST (FAST) score for the non-invasive identification of patients with non-alcoholic steatohepatitis with significant activity and fibrosis: a prospective derivation and global validation study. Lancet Gastroenterol Hepatol 2020;5:362–373.

[18] Bedossa P, FLIP Pathology Consortium. Utility and appropriateness of the fatty liver inhibition of progression (FLIP) algorithm and steatosis, activity, and fibrosis (SAF) score in the evaluation of biopsies of nonalcoholic fatty liver disease. Hepatology 2014;60:565–575.

[19] Boursier J, Canivet CM, Costentin C, et al. Impact of type 2 diabetes on the accuracy of noninvasive tests of liver fibrosis with resulting clinical implications. Clin Gastroenterol Hepatol 2023;21:1243. 51.e1212.

[20] Papatheodoridi M, Hiriart JB, Lupsor-Platon M, et al. Refining the Baveno VI elastography criteria for the definition of compensated advanced chronic liver disease. J Hepatol 2021;74:1109–1116.

[21] Boursier J, Zarski JP, de Ledinghen V, et al. Determination of reliability criteria for liver stiffness evaluation by transient elastography. Hepatology 2013;57:1182–1191.

[22] Boursier J, Guillaume M, Leroy V, et al. New sequential combinations of non-invasive fibrosis tests provide an accurate diagnosis of advanced fibrosis in NAFLD. J Hepatol 2019;71:389–396.

[23] Sanyal AJ, Foucquier J, Younossi ZM, et al. Enhanced diagnosis of advanced fibrosis and cirrhosis in individuals with NAFLD using FibroScan-based Agile scores. J Hepatol 2023;78:247–259.

[24] Boursier J, Roux M, Costentin C, et al. Practical diagnosis of cirrhosis in non-alcoholic fatty liver disease using currently available non-invasive fibrosis tests. Nat Commun 2023;14:5219.

[25] Selvaraj EA, Mozes FE, Jayaswal ANA, et al. Diagnostic accuracy of elastography and magnetic resonance imaging in patients with NAFLD: a systematic review and meta-analysis. J Hepatol 2021;75:770–785.

[26] Noureddin M, Truong E, Gornbein JA, et al. MRI-based (MAST) score accurately identifies patients with NASH and significant fibrosis. J Hepatol 2022;76:781–787.
[27] Kim BK, Tamaki N, Imajo K, et al. Head-to-head comparison between MEFIB, MAST, and FAST for detecting stage 2 fibrosis or higher among patients with NAFLD. J Hepatol 2022;77:1482–1490.
[28] Castera L, Garteiser P, Laouenan C, et al. Prospective head-to-head comparison of non-invasive scores for diagnosis of fibrotic MASH in patients with type 2 diabetes. J Hepatol 2024;81:195–206.
[29] Sanyal AJ, Williams SA, Lavine JE, et al. Defining the serum proteomic signature of hepatic steatosis, inflammation, ballooning and fibrosis in non-alcoholic fatty liver disease. J Hepatol 2023;78:693–703.
[30] Conte M, Franceschi C, Sandri M, et al. Perilipin 2 and age-related metabolic diseases: a new perspective. Trends Endocrinol Metab 2016;27:893–903.
[31] Angelini G, Panunzi S, Castagneto-Gissey L, et al. Accurate liquid biopsy for the diagnosis of non-alcoholic steatohepatitis and liver fibrosis. Gut 2023;72:392–403.
[32] Vali Y, Lee J, Boursier J, et al. Biomarkers for staging fibrosis and non-alcoholic steatohepatitis in non-alcoholic fatty liver disease (the LITMUS project): a comparative diagnostic accuracy study. Lancet Gastroenterol Hepatol 2023;8:714–725.
[33] Sanyal AJ, Shankar SS, Yates KP, et al. Diagnostic performance of circulating biomarkers for non-alcoholic steatohepatitis. Nat Med 2023;29:2656–2664.

Keywords: MASLD; MASH; Fibrotic MASH; Non-invasive tests.

Received 17 September 2024; received in revised form 27 January 2025; accepted 29 January 2025; Available online 4 February 2025

Journal of Hepatology, Volume *7*

Supplemental information

Non-invasive tests for fibrotic MASH for reducing screen failure in therapeutic trials

Jeanne Fichez, Thomas Mouillot, Luisa Vonghia, Charlotte Costentin, Clémence Moreau, Marine Roux, Adèle Delamarre, Sven Francque, Ming-Hua Zheng, and Jérôme Boursier

Reducing screen failure in MASH therapeutic trials using dedicated non-invasive tests for fibrotic MASH

Jeanne Fichez, Thomas Mouillot, Luisa Vonghia, Charlotte Costentin, Clémence Moreau, Marine Roux, Adèle Delamarre, Sven Francque, Ming-Hua Zheng, Jérôme Boursier

Table of contents

Table S1 2

Table S2 3

Table S3 4

Table S4 5

Table S5 6

Table S6 7

Table S7 8

Table S8 9

Table S9 10

Table S10 11

Fig. S1 12

Fig. S2 15

Fig. S3 17

Fig. S4 18

Table S1: Non-invasive tests evaluated in the study

Category		Test	Diagnostic target [a]	Variable
Blood tests	Simple	FNI (1)	Fibrotic MASH	AST, HbA1c, HDL cholesterol
		FIB4 (2)	Advanced fibrosis	Age, AST, ALT, platelets
	Specialized	MACK-3 (3)	Fibrotic MASH	AST, HOMA index, CK-18
		ELF (4)	Advanced fibrosis	Hyaluronate, TIMP1, P3NP
		Fibrotest (5)	Advanced fibrosis	Age, sex, gamma-GT, bilirubin, haptoglobin, apolipoproteinA1, α2macroglobulin
Elastography-based tests		VCTE (2)	-	Liver stiffness
		FAST (6)	Fibrotic MASH	AST, liver stiffness, CAP
		Agile3+ (7)	Advanced fibrosis	Age, sex, diabetes, AST, ALT, platelets, liver stiffness

[a] Diagnostic target for which the test was developed.

ALT: alanine aminotransferase; AST: aspartate aminotransferase; CAP: control attenuation parameter; CK-18: Cytokeratin 18; Gamma-GT: gamma-glutamyl transferase; HbA1c: hemoglobin A1C; HDL: high-density lipoprotein; HOMA index: homeostatic model assessment index; MASH: metabolic dysfunction - associated steatohepatitis; P3NP: procollagen type III N-terminal peptide; TIMP1: tissue inhibitor of metalloproteinase-1; VCTE: vibration controlled transient elastography (Fibroscan device, Echosens, Paris, France)

References

1. Tavaglione F, et al. Clin Gastroenterol Hepatol 2023;21:1523-1532 e1521.
2. Mozes FE, et al. Gut 2022;71:1006-1019.
3. Boursier J, et al. Aliment Pharmacol Ther 2018;47:1387-1396.
4. Vali Y, et al. J Hepatol 2020;73:252-262.
5. Vali Y, et al. J Clin Med 2021;10.
6. Newsome PN, et al. Lancet Gastroenterol Hepatol 2020;5:362-373.
7. Sanyal AJ, et al. J Hepatol 2023;78:247-259.

Table S2: Study analyses

Group	Patients (n)	Fibrosis tests available	Analyses
Study population	1,005	FNI, FIB4	• Direct comparison of two simple blood fibrosis tests: FNI (developed for fibrotic MASH) and FIB4 (developed for advanced fibrosis)
Elastography group	817	FNI, FIB4<br>VCTE<br>FAST, Agile3+	• Direct comparison of three elastography-based tests: VCTE, FAST (developed for fibrotic MASH), and Agile3+ (developed for advanced fibrosis)<br>• Evaluation of the added value of elastography-based tests (VCTE, FAST, Agile3+) compared to simple blood tests (FNI, FIB4)
Specialized blood tests group	545	FNI, FIB4<br>MACK-3<br>ELF, Fibrotest	• Direct comparison of three specialized blood fibrosis tests: MACK-3 (developed for fibrotic MASH), ELF (developed for advanced fibrosis), and Fibrotest (developed for advanced fibrosis)<br>• Evaluation of the added value of specialized blood tests (MACK-3, ELF, Fibrotest) compared to simple blood tests (FNI, FIB4)

Table S3: AUROC for the diagnosis of fibrotic MASH and the diagnosis of advanced fibrosis in the 747 patients with reliable VCTE

	Elastography group with reliable VCTE result (n = 747)	
**Diagnostic target:**	**Fibrotic MASH**	**Advanced fibrosis**
FNI	0.689 (0.651-0.727)	0.689 (0.648-0.730)
FIB4	0.663 (0.624-0.702)	0.792 (0.758-0.826)
FAST	0.759 (0.725-0.793)	0.759 (0.723-0.796)
VCTE	0.722 (0.685-0.759)	0.825 (0.794-0.857)
Agile3+	0.701 (0.664-0.739)	0.859 (0.831-0.859)
P value (comparison):		
FNI *vs.* FIB4	0.554	<0.001
FNI *vs.* FAST	<0.001	<0.001
FNI *vs.* VCTE	0.469	<0.001
FNI *vs.* Agile 3+	0.618	<0.001
FIB4 *vs.* FAST	<0.001	0.518
FIB4 *vs.* VCTE	0.056	0.647
FIB4 *vs.* Agile3+	0.061	<0.001
FAST *vs.* VCTE	0.108	<0.001
FAST *vs.* Agile3+	0.038	<0.001
Agile3+ *vs.* VCTE	0.551	0.047

MASH: metabolic dysfunction-associated steatohepatitis; VCTE: vibration controlled transient elastography

Table S4: Internal validation on 1,000 bootstrap samples of the optimized thresholds of FNI, FIB4, VCTE, Agile3+, ELF and Fibrotest for fibrotic MASH

Results correspond to mean percentages with 95% confidence intervals in parentheses.

Group	Test	Threshold	Patients (%) [a]	Se (%)	Spe (%)	NPV (%)	PPV (%)	Grey zone (%) [b]
Study population (n = 1,055)	FNI	<0.23	24.5 (24.4, 24.5)	89.8 (89.7, 89.8)	34.6 (34.6, 34.7)	82.5 (82.4, 82.6)	49.6 (49.5, 49.7)	58.4 (58.4, 58.5)
		>0.80	17.1 (17.1, 17.2)	26.2 (26.1, 26.3)	89.4 (89.3, 89.4)	62.8 (62.8, 62.9)	63.9 (63.7, 64.0)	
	FIB4	<0.80	21.4 (21.3, 21.4)	89.3 (89.3, 89.4)	29.1 (29.0, 29.1)	79.2 (79.8, 79.3)	47.4 (47.4, 47.5)	62.3 (62.2, 62.4)
		>2.30	16.3 (16.3, 16.4)	24.5 (24.5, 24.6)	89.6 (89.5, 89.6)	62.4 (62.3, 62.4)	62.8 (62.6, 62.9)	
Elastography group (n = 817)	VCTE	<6.0	29.6 (29. 6, 29.7)	89.0 (88.9, 89.1)	42.7 (42.7, 42.9)	84.6 (84.5, 84.7)	52.3 (52.2, 52.4)	54.9 (54.8, 54.9)
		>15.4	15.5 (15.5, 15.6)	23.9 (23.8, 24.0)	90.4 (90.3, 90.4)	62.7 (62.7, 62.8)	63.6 (63.5, 63.8)	
	Agile3+	<0.102	27.5 (27.4, 27.5)	88.1 (88.0, 88.1)	38.4 (38.3, 38.5)	82.0 (81.9, 82.1)	50.2 (50.1, 50.3)	56.1 (56.0, 56.2)
		>0.844	16.4 (16.4, 16.5)	25.1 (25.0, 25.2)	89.7 (89.7, 89.8)	62.9 (62.9, 63.0)	63.3 (63.1, 63.5)	
Specialized blood tests group (n = 545)	ELF	<8.6	23.2 (23.1, 23.2)	88.7 (88.6, 88.8)	33.1 (33.0, 33.3)	77.8 (77.6, 78.0)	52.7 (52.6, 52.8)	57.8 (57.7, 57.9)
		>10.4	19.1 (19.0, 19.2)	29.3 (29.2, 29.4)	89.5 (89.4, 89.6)	60.1 (60.0, 60.2)	70.1 (69.9, 70.3)	
	Fibrotest	<0.14	15.4 (15.3, 15.5)	89.9 (89.8, 90.0)	19.9 (19.8, 20.0)	70.2 (70.0, 70.4)	48.5 (48.4, 48.6)	72.5 (72.4, 72.6)
		>0.71	12.1 (12.1, 12.2)	15.2 (15.1, 15.3)	90.5 (90.4, 90.6)	56.0 (55.9, 56.1)	57.4 (57.2, 57.7)	

[a] Patients included in the interval defined by the diagnostic threshold; [b] Rate of patients in the interval between the two diagnostic thresholds.
Se: sensitivity; Spe: specificity; NPV: negative predictive value; PPV: positive predictive value

Table S5: Accuracy of non-invasive tests for the diagnosis of fibrotic MASH with optimized thresholds, as a function of the prevalence of fibrotic MASH

Group	Test	Threshold		Fibrotic MASH prevalence					
				5%	10%	20%	30%	40%	50%
Study population (n = 1,055)	FNI	<0.23	% Patients (NPV)	33.8 (99)	32.5 (97)	30.0 (93)	27.5 (89)	25.0 (84)	22.5 (78)
		0.23-0.80	% Patients	54.5	55.0	56.0	57.0	58.0	59.0
		>0.80	% Patients (PPV)	11.8 (11)	12.5 (21)	14.0 (37)	15.5 (50)	17.0 (61)	18.5 (70)
	FIB-4	<0.80	% Patients (NPV)	28.1 (98)	27.2 (96)	25.4 (91)	23.6 (86)	21.8 (80)	20.0 (73)
		0.80-2.30	% Patients	61.2	61.3	61.6	61.9	62.2	62.5
		>2.30	% Patients (PPV)	10.8 (12)	11.5 (22)	13.0 (38)	14.5 (52)	16.0 (63)	17.5 (71)
Elastography group (n = 817)	FAST	≤0.35	% Patients (NPV)	50.8 (99)	48.8 (97)	44.7 (94)	40.7 (91)	36.6 (86)	32.6 (81)
		0.35-0.67	% Patients	34.8	35.3	36.4	37.4	38.5	39.5
		≥0.67	% Patients (PPV)	14.4 (15)	15.9 (27)	18.9 (45)	21.9 (59)	24.9 (69)	27.9 (77)
	VCTE	<6.0	% Patients (NPV)	41.4 (99)	39.8 (97)	36.6 (94)	33.4 (90)	30.2 (85)	27.0 (80)
		6.0-15.4	% Patients	47.9	48.8	50.6	52.4	54.2	56.0
		>15.4	% Patients (PPV)	10.7 (11)	11.4 (21)	12.8 (38)	14.2 (51)	15.6 (62)	17.0 (71)
	Agile3+	<0.102	% Patients (NPV)	36.7 (98)	35.4 (97)	32.8 (93)	30.2 (88)	27.6 (83)	25.0 (76)
		0.102-0.844	% Patients	52.6	53.1	54.2	55.3	56.4	57.5
		>0.844	% Patients (PPV)	10.8 (12)	11.5 (22)	13.0 (38)	14.5 (52)	16.0 (63)	17.5 (71)
Specialized blood tests group (n = 545)	MACK-3	<0.135	% Patients (NPV)	38.2 (99)	36.6 (98)	33.2 (96)	29.9 (94)	26.5 (90)	23.2 (86)
		0.135-0.549	% Patients	44.8	45.0	45.5	45.9	46.4	46.8
		>0.549	% Patients (PPV)	17.0 (13)	18.4 (24)	21.3 (42)	24.2 (55)	27.1 (66)	30.1 (74)
	ELF	<8.6	% Patients (NPV)	31.9 (98)	30.8 (96)	28.6 (92)	26.4 (88)	24.2 (82)	22.0 (75)
		8.6-10.4	% Patients	57.2	57.3	57.6	57.9	58.2	58.5
		>10.4	% Patients (PPV)	11.0 (13)	11.9 (24)	13.8 (42)	15.7 (55)	17.6 (66)	19.5 (74)
	Fibrotest	<0.14	% Patients (NPV)	19.5 (97)	19.0 (95)	18.0 (89)	17.0 (82)	16.0 (75)	15.0 (67)
		0.14-0.71	% Patients	71.2	71.4	71.8	72.2	72.6	73.0
		>0.71	% Patients (PPV)	9.3 (8)	9.6 (16)	10.2 (29)	10.8 (42)	11.4 (53)	12.0 (63)

NPV: negative predictive value; PPV: positive predictive value

Table S6: Accuracy of non-invasive tests for the diagnosis of fibrotic MASH with optimized thresholds in the 747 patients with reliable VCTE

Group	Test	Threshold	Patients (%) [a]	Se (%)	Spe (%)	NPV (%)	PPV (%)	Grey zone (%) [b]
Elastography group (n = 747)	VCTE	<6.0	30.7	88	43	84	52	55.0
		>15.4	14.3	22	91	63	62	
	Agile3+	<0.102	22.1	88	39	83	50	57.0
		>0.844	14.9	22	90	63	59	

[a] Patients included in the interval defined by the diagnostic threshold
[b] Rate of patients in the interval between the two diagnostic thresholds
Se: sensitivity; Spe: specificity; NPV: negative predictive value; PPV: positive predictive value; VCTE: vibration controlled transient elastography

Table S7: Prevalence of fibrotic MASH in the diagnostic zones of non-invasive tests when they are used within the grey zone of FAST or MACK-3

Grey zone	Fibrosis test	Rule-out zone		Grey zone	Rule-in zone	
		Threshold	Fibrotic MASH (%)	Fibrotic MASH (%)	Threshold	Fibrotic MASH (%)
FAST	FNI	<0.23	33	52	>0.80	35
	FIB4	<0.80	34	51	>2.30	57
	VCTE	<6.0	28	55	>15.4	44
	Agile3+	<0.102	28	54	>0.844	67
	MACK-3	<0.135	20	48	>0.549	47
	ELF	<8.6	36	61	>10.4	64
	Fibrotest	<0.14	48	59	>0.71	59
MACK-3	FNI	<0.23	25	45	>0.80	42
	FIB4	<0.80	23	46	>2.30	50
	FAST	≤0.35	16	48	≥0.67	52
	VCTE	<6.0	19	48	>15.4	47
	Agile3+	<0.102	21	46	>0.844	52
	ELF	<8.6	28	52	>10.4	67
	Fibrotest	<0.14	40	52	>0.71	41

Table S8: FAST / Agile3+ and MACK-3 / ELF algorithms for the diagnosis of fibrotic MASH

	MACK3 <0.135	MACK3 0.135-0.549			MACK3 >0.549
		ELF <8.6	ELF 8.6-10.4	ELF >10.4	
All (n)	134	60	161	33	157
No fibrotic NASH (n)	118	43	78	11	46
Fibrotic NASH (n)	16	17	83	22	111

	FAST ≤ 0.35	FAST 0.35-0.67			FAST ≥ 0.67
		Agile3+ <0.102	Agile 3+ 0.102-0.844	Agile 3+ > 0.844	
All (n)	295	92	187	36	207
No fibrotic NASH (n)	253	66	86	12	62
Fibrotic NASH (n)	42	26	101	24	145

Table S9: Detailed histological characteristics of the patients as a function of the diagnostic intervals of the FAST / Agile3+ algorithm

Green boxes correspond to the patients well classified for fibrotic MASH, and grey boxes to those misclassified for fibrotic MASH.

Fibrosis stage	Rule out interval (n=387)			Grey zone (n=187)			Rule in interval (n=243)		
	No MASH	MASH & MAS = 3	MASH & MAS ≥4	No MASH	MASH & MAS = 3	MASH & MAS ≥4	No MASH	MASH & MAS = 3	MASH & MAS ≥4
0	85	18	27	9	1	6	3	0	2
1	46	26	58	14	9	18	6	1	21
2	20	19	51	5	8	57	4	6	46
3	12	7	17	5	8	36	7	4	87
4	1	0	0	2	1	8	11	9	36

MASH: metabolic associated-dysfunction steatohepatitis; MAS: MASLD activity score

Table S10: Detailed histological characteristics of the patients as a function of the diagnostic interval of the MACK-3 / ELF algorithm

Green boxes correspond to the patients well classified for fibrotic MASH, and grey boxes to those misclassified for fibrotic MASH.

Fibrosis stage	Rule out interval (n=194)			Grey zone (n=161)			Rule in interval (n=190)		
	No MASH	MASH & MAS = 3	MASH & MAS ≥4	No MASH	MASH & MAS = 3	MASH & MAS ≥4	No MASH	MASH & MAS = 3	MASH & MAS ≥4
0	35	0	6	8	0	6	1	0	1
1	41	15	15	7	8	12	3	4	11
2	17	19	26	8	9	47	2	8	35
3	5	6	6	9	10	32	8	8	69
4	2	0	1	1	0	4	7	4	29

MASH: metabolic associated-dysfunction steatohepatitis; MAS: MASLD activity score

Fig. S1: Observed probability of fibrotic MASH as a function of tests results

The LOWESS curve by weighted least squares (in red) was used to determine the average trend of relationships between the tests results and the presence of fibrotic MASH (Y axis on the right).

Yellow bars represent the number of patients included in each test interval (Y axis on the left).

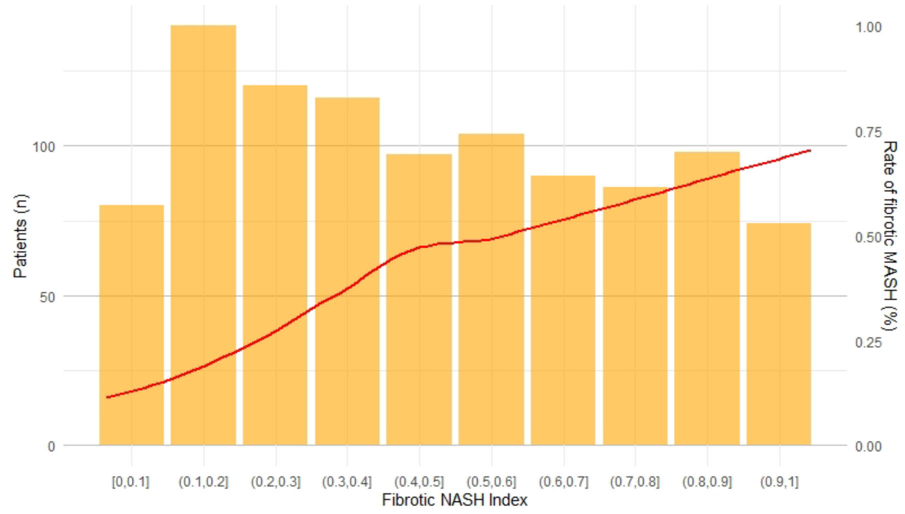

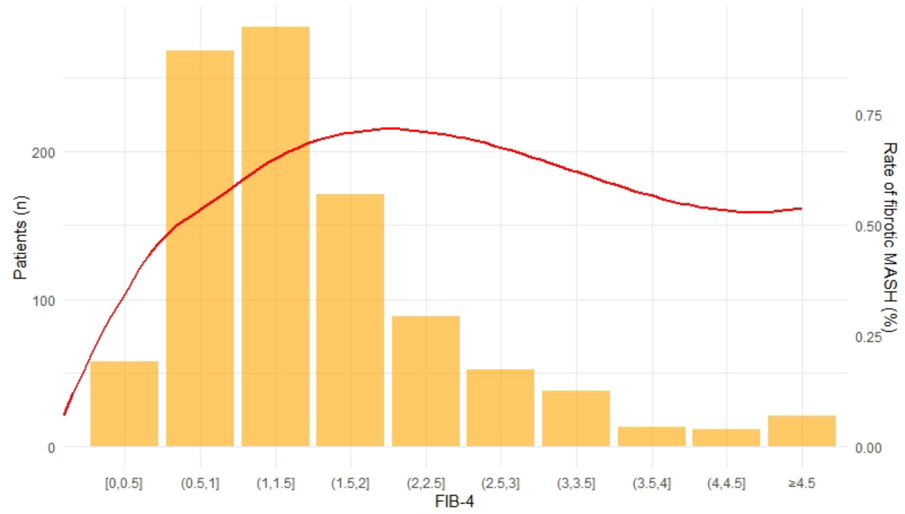

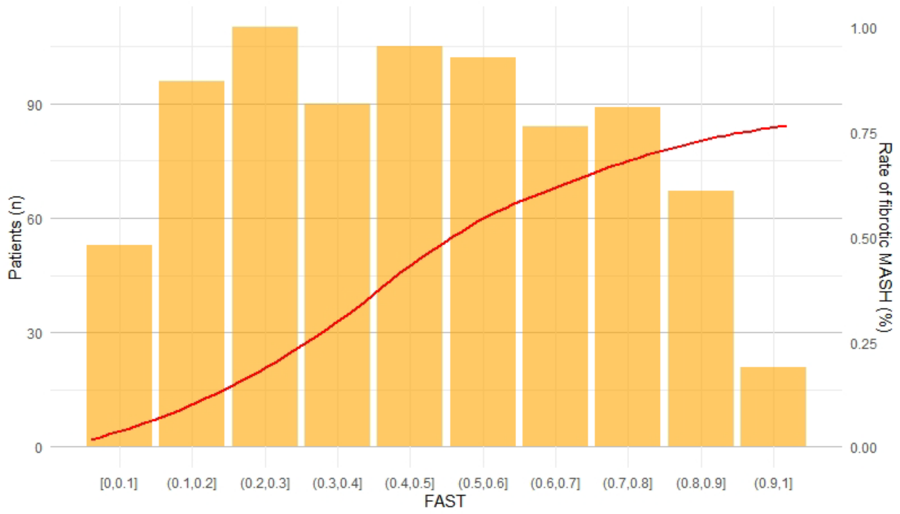

Patients (n)
Rate of fibrotic MASH (%)
0
30
60
90
0.00
0.25
0.50
0.75
1.00
[0,0.1]
(0.1,0.2]
(0.2,0.3]
(0.3,0.4]
(0.4,0.5]
(0.5,0.6]
(0.6,0.7]
(0.7,0.8]
(0.8,0.9]
(0.9,1]
FAST

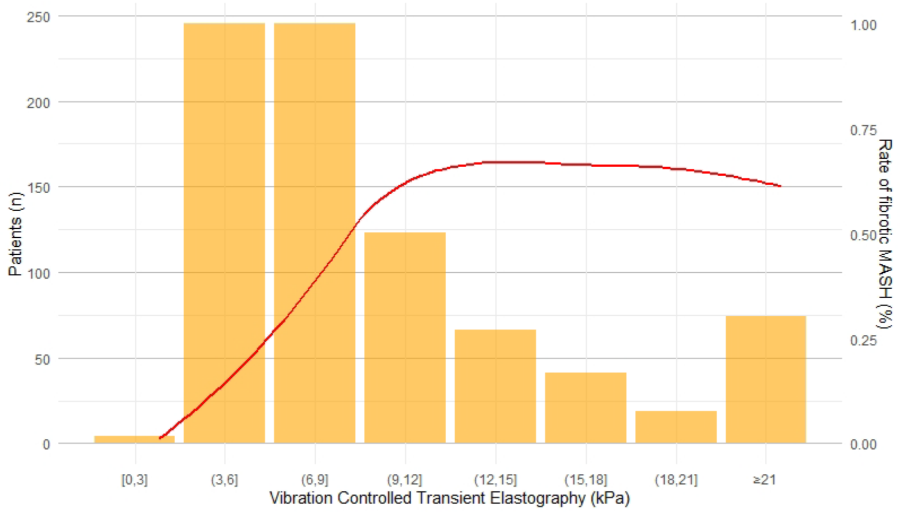

Patients (n)
Rate of fibrotic MASH (%)
0
50
100
150
200
250
0.00
0.25
0.50
0.75
1.00
[0,3]
(3,6]
(6,9]
(9,12]
(12,15]
(15,18]
(18,21]
≥21
Vibration Controlled Transient Elastography (kPa)

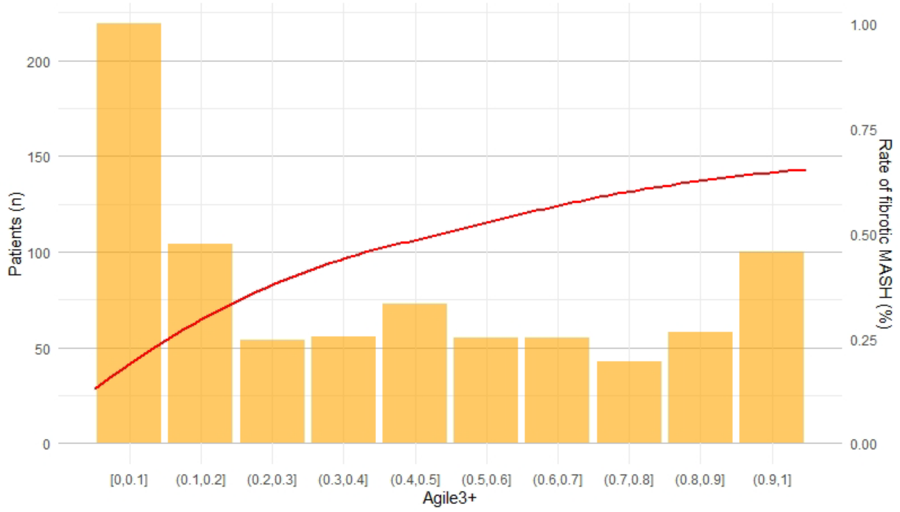

Patients (n)
Rate of fibrotic MASH (%)
0
50
100
150
200
0.00
0.25
0.50
0.75
1.00
[0,0.1]
(0.1,0.2]
(0.2,0.3]
(0.3,0.4]
(0.4,0.5]
(0.5,0.6]
(0.6,0.7]
(0.7,0.8]
(0.8,0.9]
(0.9,1]
Agile3+

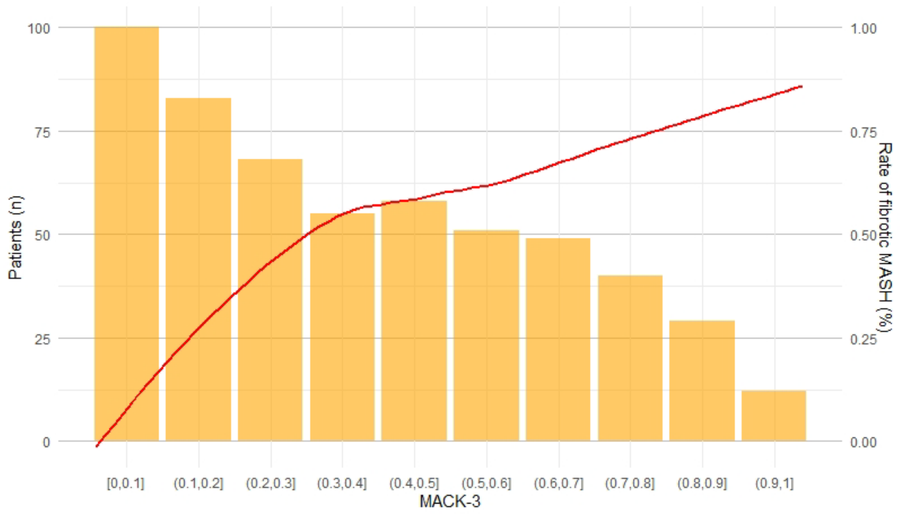

Patients (n)
Rate of fibrotic MASH (%)
[0,0.1]
(0.1,0.2]
(0.2,0.3]
(0.3,0.4]
(0.4,0.5]
(0.5,0.6]
(0.6,0.7]
(0.7,0.8]
(0.8,0.9]
(0.9,1]
MACK-3

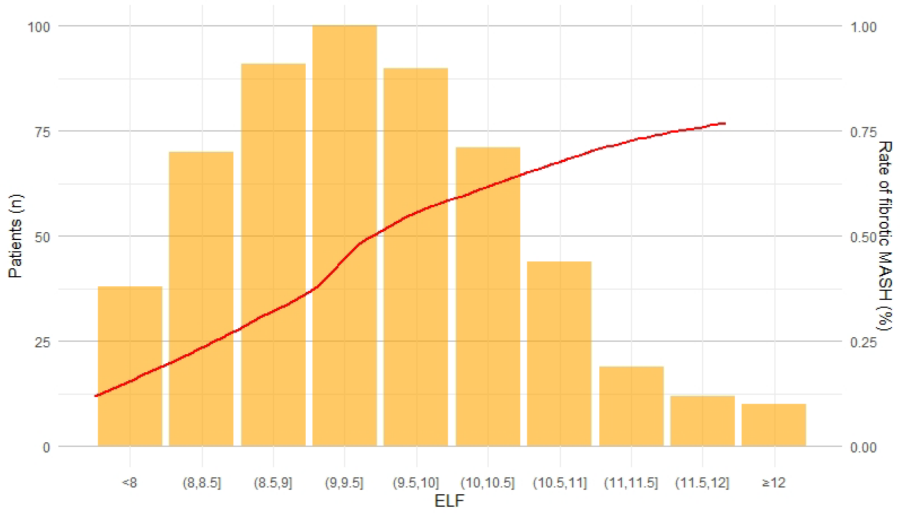

Patients (n)
Rate of fibrotic MASH (%)
<8
(8,8.5]
(8.5,9]
(9,9.5]
(9.5,10]
(10,10.5]
(10.5,11]
(11,11.5]
(11.5,12]
≥12
ELF

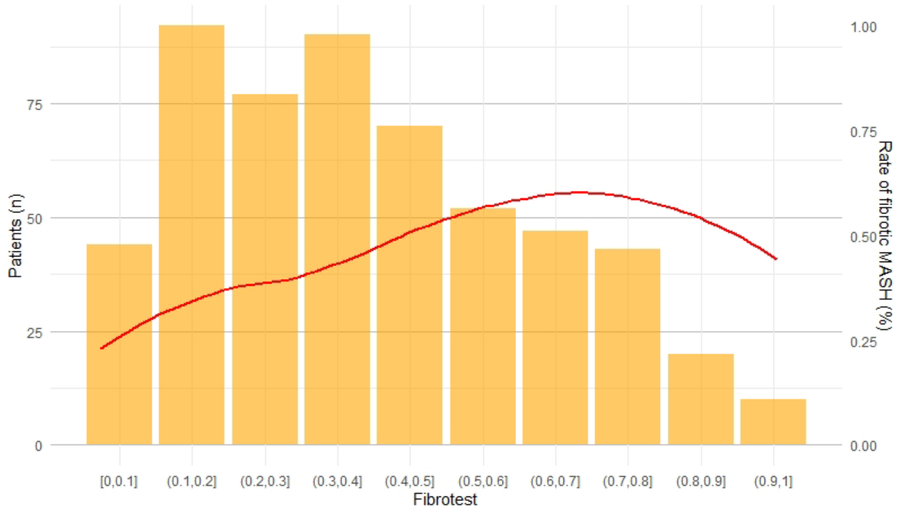

Patients (n)
Rate of fibrotic MASH (%)
[0,0.1]
(0.1,0.2]
(0.2,0.3]
(0.3,0.4]
(0.4,0.5]
(0.5,0.6]
(0.6,0.7]
(0.7,0.8]
(0.8,0.9]
(0.9,1]
Fibrotest

Fig. S2: Results (mean with 95% confidence interval) of non-invasive tests between patients with or without fibrotic MASH, in each of the three diagnostic zones of FAST

FNI:

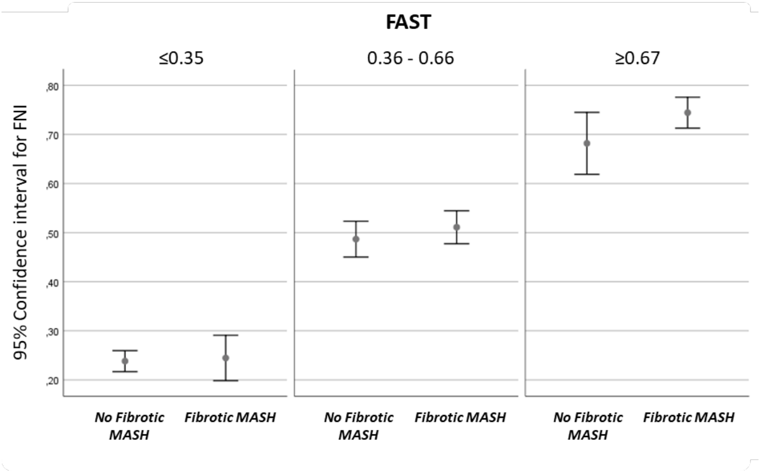

FIB4:

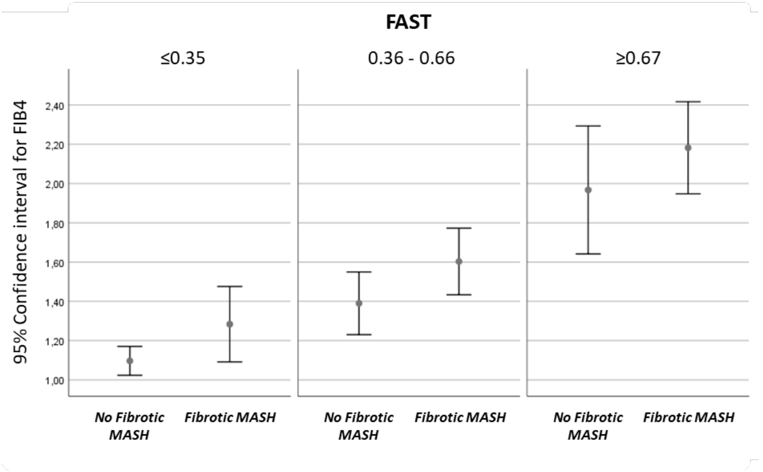

VCTE:

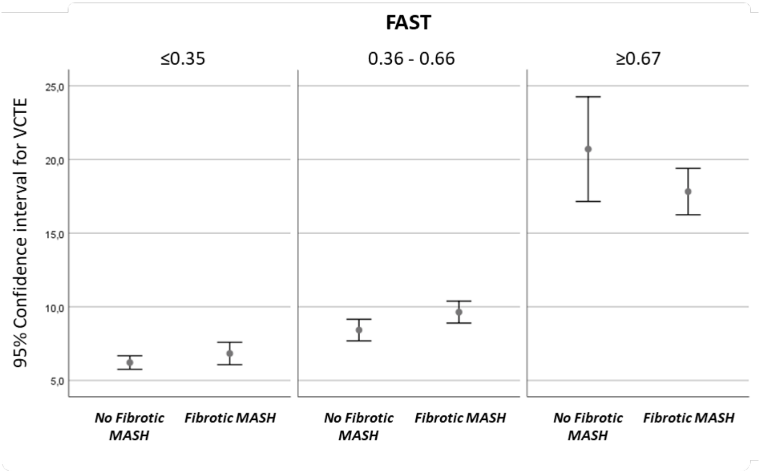

Agile3+:

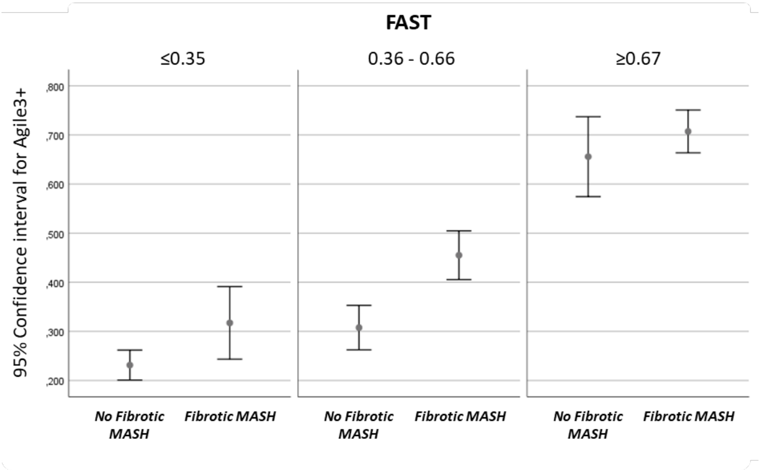

MACK-3:

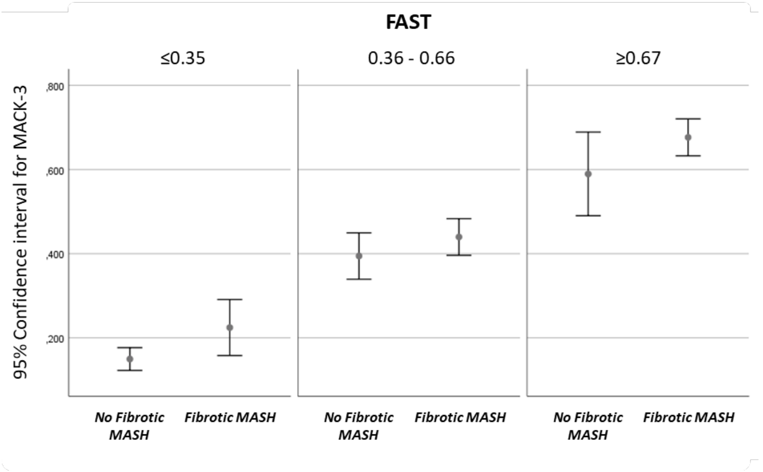

ELF:

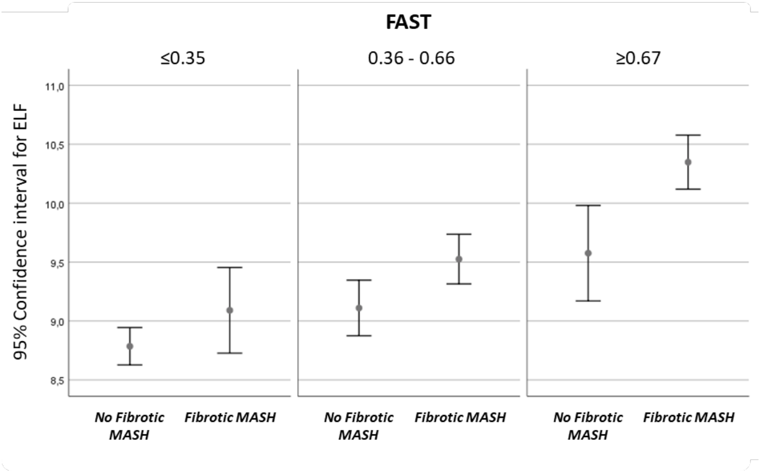

Fibrotest:

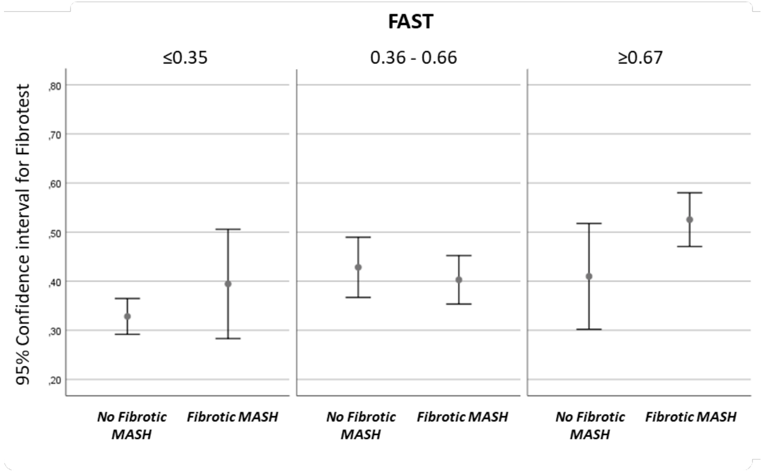

Fig. S3: Results for the FAST / Agile3+ algorithm in the 747 patients with reliable VCTE

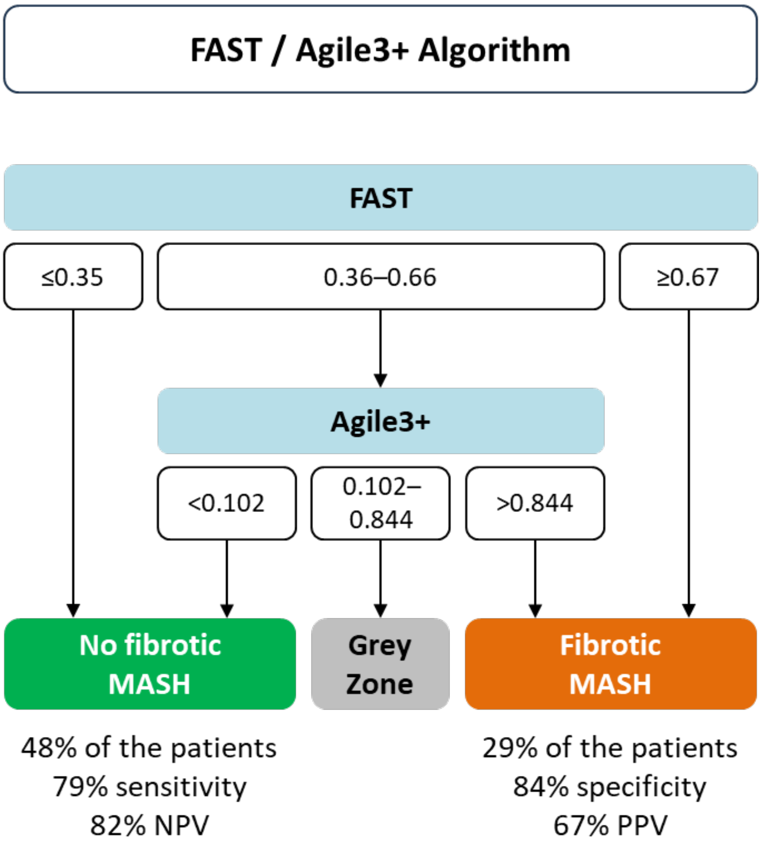

Fig. S4: Results (mean with 95% confidence interval) of non-invasive tests between patients with or without fibrotic MASH, in each of the three diagnostic zones of MACK-3

FNI:

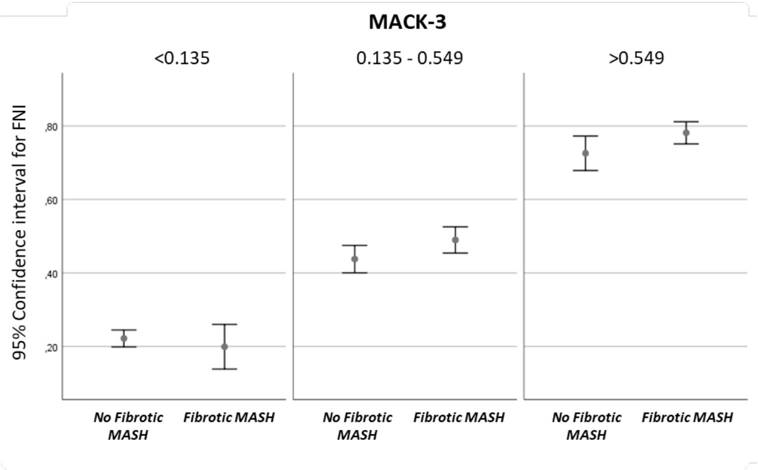

FIB4:

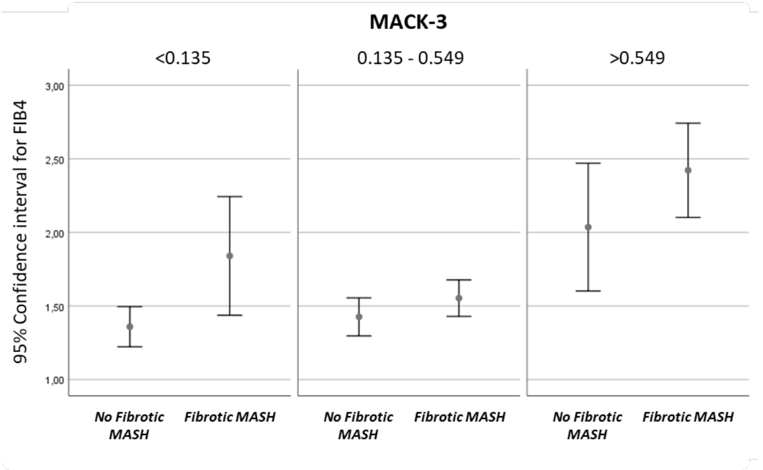

FAST:

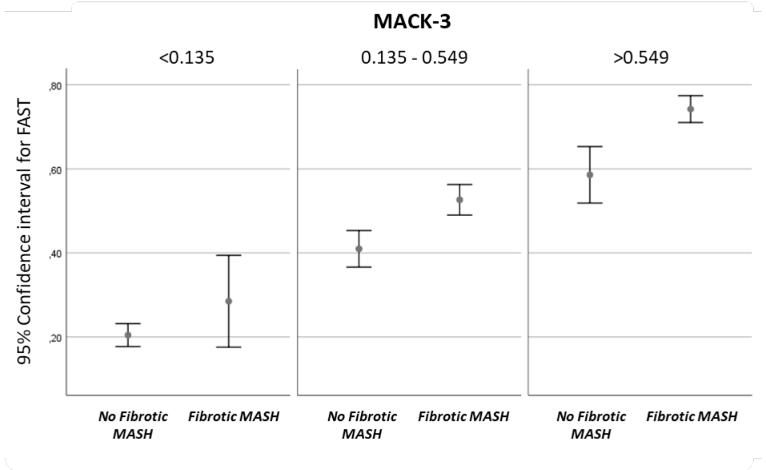

VCTE:

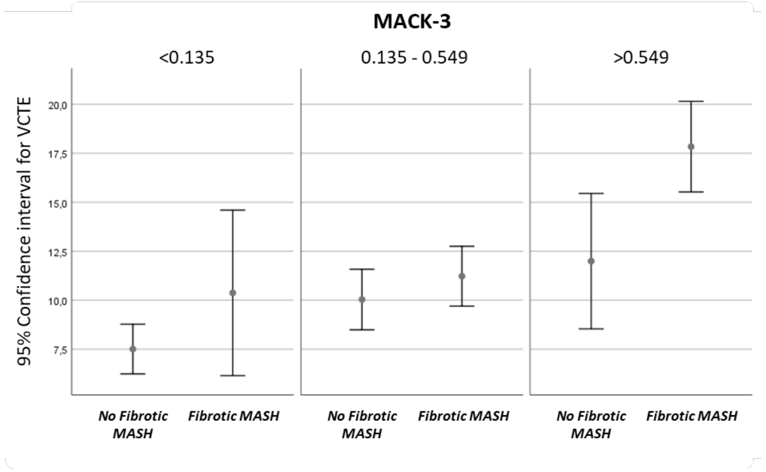

Agile3+:

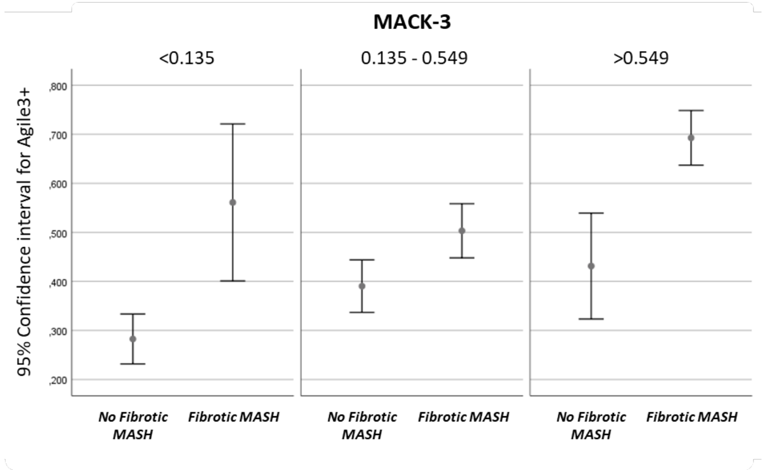

ELF:

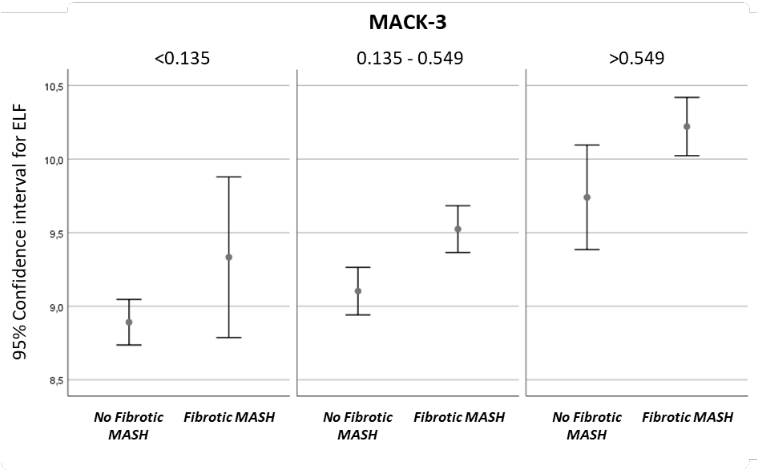

Fibrotest:

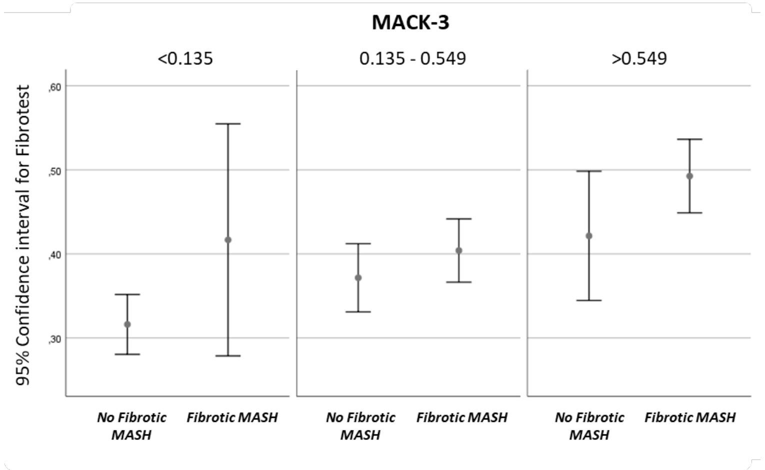

Supplement: Multimedia component 5 [file mmc5.pdf]
